# Green Synthesis of *Terminalia ferdinandiana* Exell-Mediated Silver Nanoparticles and Evaluation of Antibacterial Performance

**DOI:** 10.3390/biom14121516

**Published:** 2024-11-27

**Authors:** W. Hansi S. Alwis, Vinuthaa Murthy, Hao Wang, Roshanak Khandanlou, Pappu Kumar Mandal

**Affiliations:** 1 Faculty of Science and Technology, Charles Darwin University, Darwin, NT 0810, Australia; hansi.alwis@cdu.edu.au (W.H.S.A.); hao.wang@cdu.edu.au (H.W.); roshanak_bch@yahoo.com (R.K.); 2 Menzies School of Health Research, Darwin, NT 0810, Australia; pappukmandal@gmail.com

**Keywords:** Kakadu plum, green nanoparticles, antioxidant, MIC, LC-MS, Australian native plants

## Abstract

This study uses a novel method in which extracts from different parts of a single plant are used to synthesize well-defined silver nanoparticles (AgNPs) to address the lack of capping agents in certain plant extracts. We focused on synthesizing AgNPs with enhanced biomedical activity using aqueous leaves and fruit extracts of *Terminalia ferdinandiana* Exell, a plant native to northern Australia that is known for its high phenolic content and associated health benefits. The impact of using parameters such as the Ag^+^ ion-to-extract ratio and pH on AgNP synthesis was examined. The formation of AgNPs was confirmed using UV–visible spectrophotometry, transmission electron microscopy, and dynamic light scattering. The AgNPs synthesized at a pH of 8 and 1:25 Ag^+^/extract ratio exhibited the lowest particle size and polydispersity index. The AgNPs synthesized with leaf extract (AgKL) were monodisperse and exhibited a smaller hydrodynamic diameter (37 nm) compared to the fruit extract nanoparticles (AgKP), which were polydisperse and larger (147 nm). Phytochemicals in *T. ferdinandiana* aqueous leaf extract act as effective capping and stabilizing agents, enabling the synthesis of small-sized and homogenous AgNPs, which the fruit extract alone could not achieve. The in vitro bioactivity was evaluated using antioxidant and antibacterial assays and compared with the crude extract. Both the AgNPs and *T. ferdinandiana* extracts demonstrated strong 2,2 diphenyl-1-picrylhydrazyl radical scavenging activity. However, only AgKL showed excellent antibacterial activity against Gram-negative and Gram-positive bacteria based on minimum inhibitory and bactericidal results. Mixing 50% leaf extract with fruit extract resulted in well-stabilized NPs (AgKPL) with a hydrodynamic diameter of 33.4 nm and superior antibacterial properties. These results indicate that AgKL and AgKPL have significant potential for pharmaceutical and biomedical applications.

## 1. Introduction

In recent years, many researchers have turned their attention to nanosystems due to their specific features of high reactivity and small size [1], as they tend to have remarkable applications in targeted delivery in the medicinal and agricultural fields [2,3]. Furthermore, metal nanoparticles (NPs), which are mainly synthesized using noble metals such as silver, have gained significant interest because of their high stability, durability, reactivity, and, most importantly, antibacterial and antifungal properties [4]. Previous studies have demonstrated that the antibacterial activity of silver nanoparticles (AgNPs) is firmly size dependent [5,6]; for instance, smaller nanoparticles have a greater capacity for penetrating the bacterial cell wall. Also, smaller particle sizes provide increased surface area even at low concentrations, allowing them to make contact with the vast surface area of the bacteria, thereby increasing the reactivity [5,6,7]. Silver nanoparticles are being used as antimicrobial agents in the production of medical devices [8], wound dressings [9], cosmetics [10,11], textiles [12,13,14], home water purification systems [15], and household appliances [16].

Nanoparticles are traditionally synthesized using various chemical and physical methods, including a recently developed eco-friendly approach called green synthesis [17], which aims to reduce the hazardous chemical wastes generated during synthesis by using environmentally friendly materials, non-toxic chemicals, and safe solvents in the synthesis protocol [18].

The current literature details the plant extract-mediated synthesis of metal NPs with a shape, size, and stability suitable for biological and medical applications [2,19]. The role of phytochemicals in aqueous plant extracts is important for their green synthesis. They act as reducing, stabilizing, and capping agents in the green synthesis of NPs and have unique properties that are predominantly applied in the pharmaceutical and medical sectors. Silver nanoparticles have a strong oxidative activity with the potential to release silver (Ag) ions that could induce cytotoxicity, genotoxicity, immune responses, and even cell death [20]. However, during green synthesis, the biogenic compounds present in plant material form a stabilizing layer around the NPs, promoting biocompatibility [21], mitigating potential adverse effects [22], and offering significant advantages for medical applications like wound healing and drug delivery, where maintaining healthy tissue integrity is crucial. 

The literature on the green synthesis of AgNPs using different plant extracts has reported effective antimicrobial activity [23,24]. Green-synthesized AgNPs using *Aloe maculata* [25] extract exhibited better antibacterial activity against Gram-positive and Gram-negative bacteria than the extract alone. In vitro studies of green-synthesized AgNPs of leaf extracts of *Argemone mexicana* [26] and *Chenopodium murale* [27] found that they were highly effective as antibacterial agents. Silver nanoparticles synthesized using *Skimmia laureola* [28] leaf extract showed bacteriostatic effects against Gram-positive and Gram-negative human pathogenic bacteria, such as *Escherichia coli*, *Klebsiella pneumoniae*, *Pseudomonas aeruginosa*, *Proteus vulgaris*, and *Staphylococcus aureus*.

*Terminalia ferdinandiana* Exell, commonly known as the Kakadu plum, Billy Goat plum, and Bush plum, belongs to the Combretaceae family and is native to Australia. It is found in subtropical woodlands of the Northern Territory (NT) and Queensland (Qld) and in the Kimberley regions of Western Australia [29]. Kakadu plum fruit has been utilized as a food source and in traditional medicine, while the bark and leaves have been employed to treat colds, the flu, and various skin conditions, including skin disorders, fungal infections, and bacterial infections, by Indigenous Australians for thousands of years [29]. *T. ferdinandiana* fruit has a high level of vitamin C, which is 50-times (by wet weight) higher than the vitamin C in oranges [30] and approximately 900-times (by dry weight) higher than that in blueberries [31]. Although the high level of ascorbic acid in *T. ferdinandiana* fruit extract is responsible for its antioxidant potential, it also contains other polyphenolic antioxidants [31] that exhibit in vitro antimicrobial, antioxidant, anti-inflammatory, and anticancer properties [32,33]. *T. ferdinandiana* fruit pulp extracts have broad-spectrum antibacterial activity, and the antibacterial activity toward Gram-positive bacteria is more significant than toward Gram-negative bacteria [34]. *T. ferdinandiana* leaves also contain various bioactive flavonoids, phenolic acids, and tannins, which possess numerous health-enriching properties [35]. Leaf extracts from *T. ferdinandiana* exhibited potent inhibitory activity against bacterial triggers of several autoimmune inflammatory diseases initiated by high tannin content [35]. Moreover, extracts of *T. ferdinandiana* leaves using different solvents inhibit the growth of axillary and plantar malodor-producing bacteria [36]. In addition, extracts of *T. ferdinandiana* from both fruit and leaves showed considerable in vitro antimicrobial properties against common foodborne bacteria, such as *S. aureus*, *Bacillus cereus*, *Listeria monocytogenes*, and *P. aeruginosa* [37]. A recent study on bio-synthesized zinc oxide NPs from *T. ferdinandiana* fruit extract showed an enhanced effect on lung cancer and anti-inflammatory activities [38]. However, there is no literature on the synthesis and application of AgNPs conjugated with *T. ferdinandiana* leaf or fruit extracts. 

Incorporating active constituents into suitable metallic NPs can significantly enhance the targeted delivery of active components and combination therapy. Furthermore, different parts of the same plant contain diverse phytochemicals, which may exhibit unique chemical properties that influence nanoparticle formation. This work focused on studying the role of phytochemicals, which are present in various parts of the same plant, in the synthesis of NPs and investigating the potential to increase the efficacy of *T. ferdinandiana* leaf and fruit extract medicinal properties by conjugating them with AgNPs. We also explored the optimal conditions for synthesizing stable and homogeneous AgNPs and reported their antibacterial activity against both Gram-positive and Gram-negative human pathogenic bacteria.

## 2. Materials and Methods

### 2.1. Materials

Fresh leaves and fruits of *T. ferdinandiana* were collected from Charles Darwin University (CDU), NT (12°36′91″ S, 130°86′68″ E), Australia. The voucher specimen is deposited at the Northern Territory Herbarium in Darwin, NT (D0293019 [Weerakkodige, H.A. *s.n*.]). Silver nitrate (AgNO_3_) and AR-grade methanol were purchased from Sigma-Aldrich, Victoria (VIC), Australia, and 2,2-diphenyl-1-picrylhydrazyl (DPPH) was purchased from Thermo Fisher Scientific, Scoresby, VIC, Australia. The bacterial strains *S. aureus* (ATCC 25923), *E. coli* (ATCC 25922), *P. aeruginosa* (ATCC 27853), and *Proteus mirabilis* (ATCC 12453) were provided by the Menzies School of Health Research, NT, Australia. Horse Blood Agar (HBA) Columbia plates, Mueller–Hinton agar (MHA) plates, and Mueller–Hinton Broth (MHB) (Oxoid, CM0405) were purchased from Thermo Fisher Scientific, VIC, Australia.

### 2.2. Preparation of the Extracts

The collected leaves and fruit of *T. ferdinandiana* were washed with high-purity water (HPW) until no foreign materials remained. Fresh plum flesh equal to 5.0 g of dry powder plums was chopped and stirred with 100.0 mL of HPW at 60 °C for 5 min. Similarly, the fresh leaves, equal to 5.0 g of dry powder leaf, were finely cut and stirred with 100.0 mL of HPW at 60 °C for 5 min, followed by 3 h of shaking at 240 rpm at room temperature (23 °C). Both extracts were filtered through Whatman No. 1 filter paper under vacuum followed by 0.45 µm filtering. The filtrate was stored at 4 °C for use in subsequent experiments in this study. 

### 2.3. Liquid Chromatography–Mass Spectroscopy Analysis

For liquid chromatography–mass spectroscopy (LC-MS) analysis, the extracts were dissolved in HPW at a concentration of 5 mg mL^−1^ and filtered through a 0.2 μm polytetrafluoroethylene (PTFE) filter for further ultra-performance liquid chromatography–mass spectrometry (UPLC-MS) analyses at Victor Chang Cardiac Research Institute, Sydney, Australia. LC-MS/MS (mass spectrometry) analysis was performed using an Agilent 1290 HPLC (high-performance liquid chromatography) system interfaced with an Agilent 6546 quadrupole time-of-flight mass spectrometer with a dual Agilent jet stream (AJS) electrospray ion source (ESI), as reported in the literature [39]. The LC separation was accomplished using an Acquity ultra-performance liquid chromatography (UPLC) high-strength silica-T3 (HSS-T3) column (1.8 μm, 2.1 × 100 mm, Waters Corporation, Milford, MA, USA) with a 2.1 × 5 mm T3 VanGuard^TM^ PreColumn (Waters Corporation, MA, USA) at 45 °C. Water and 90% acetonitrile were used as mobile phases A and B, respectively, with 0.1% formic acid and 10 mM ammonium formate. A 0.4 mL/min flow rate was applied, and the following gradient was used: 1% B inclined to 40–70% B at 11–13 min, then inclined to 100% B at 15 min and maintained for 1 min, then declined to 1% B at 17 min, followed by 2 min of conditioning. Agilent MassHunter Data Acquisition (version 11.0 build 11.022101) was used for data acquisition. The samples were analyzed in both positive and negative modes for MS1 (mass spectrum 1) acquisition; the samples were scanned from 50–1200 *m*/*z* at three spectra per second and 0 V Collision Energy (CE) using the parameters described in S1.

### 2.4. Optimization and Characterization of Silver Nanoparticles

The *T. ferdinandiana* fruit and leaf extracts were each mixed with AgNO_3_ solution separately. The solution was stirred for 30 min at room temperature and incubated overnight at room temperature in the dark. The reduction of Ag^+^ to Ag^0^ nanoparticles was followed by a color change in the solution from yellow to deep brown to dark brownish black, depending on the parameters studied. 

Two experimental parameters were evaluated to determine the ideal AgNP bio-fabrication conditions: (i) the silver ion (Ag^+^)/extract (*w*/*w*) ratio of the reaction mixture and (ii) the pH. Five ratios were evaluated by combining three different extracts (plum, leaf, and mixed plum and leaf) with 5.0 mM AgNO_3_ at ratios of 1:100, 1:50, 1:33, 1:25, and 1:20 (Ag ions/extract in mg). In order to assess the effects of the pH, the pH values of the mixture were adjusted from four to ten using 0.1 M sodium hydroxide. All AgNPs were synthesized at room temperature and were incubated in the dark for 24 h. The mean hydrodynamic diameter, polydispersity index (PDI), and ultraviolet–visible (UV-vis) spectra were used to determine the optimum conditions for the synthesis of small and homogenized NPs. Nanoparticles were also synthesized from a mixture of leaf and plum extracts using a similar method to the one used for the synthesis of AgNPs using leaf and fruit extract. 

The formation of AgNPs was confirmed using UV-vis spectroscopy (Varian, Cary 100). The UV-vis spectra of AgNPs were recorded at wavelengths of 300–800 nm, and the spectra of all extracts and AgNO_3_ were recorded at 200–800 nm. Each sample was diluted with HPW at a 1:100 ratio to keep the absorbance of AgNPs within the 0.1–1.0 range.

The mean hydrodynamic diameter, PDI, and zeta potential of the AgNPs were measured using a SZ-100 NP analyzer (Horiba, Japan). The mean hydrodynamic diameter and PDI were measured at a scattering angle of 173° at 25 °C. The zeta potential of the NPs was measured for their surface charge, which was related to their stability in solution [40]. Each sample was diluted with HPW at a 1:100 ratio to avoid inter-particle interactions and multiple scattering.

The size and shape of AgNPs were examined using a JEOL 1400 transmission electron microscope (TEM) at 120 kV at Queensland University of Technology (QUT), Brisbane, Qld, Australia. 

### 2.5. Antioxidant Activity Assay

The antioxidative capacities of the AgNPs and *T. ferdinandiana* extracts were assessed as per a previously used method [19], with modifications. Briefly, 1 mL of *T. ferdinandiana* extracts and AgNPs in methanol over the range of concentrations (0.1–0.8 mg/mL in terms of extract) were added to 1 mL of 1.0 mM methanolic solution of DPPH. After 30 min of incubation in the dark at room temperature, the absorbance was read against blank samples at 517.0 nm using a UV-vis spectrophotometer (Varian, Cary 100). The percentage inhibition of DPPH oxidation was calculated using the following equation:

$$DPPH\;scavengimg\;effect\left(\%\right)=\left(\frac{A_{control}-A_{sample}}{A_{control}}\right)\times 100$$

where $A_{control}$ is the absorbance of the DPPH solution, and $A_{sample}$ is the absorbance of the test sample. The selected concentrations correspond to the extract concentrations in the NPs, and equivalent concentrations of crude extracts were used for all tests to allow for comparison.

### 2.6. Antibacterial Activity Assay

The antibacterial activities of AgNPs and plant extracts were studied against Gram-positive (*S. aureus*) and Gram-negative (*E. coli*, *Pseudomonas aeruginosa*, and *Proteus mirabilis*) bacteria. All the bacterial strains were grown on HBA (Thermo Fisher Scientific, Perth, Australia) with a 24 h incubation at 37 °C in 5% CO_2_.

The minimum inhibitory concentrations (MICs) of the extracts and AgNPs were determined using the microplate dilution method [37], with modifications. Briefly, the plant extracts and AgNPs were diluted two-fold with HPW. Subsequently, 75 µL of extracts and AgNPs (six different concentrations of each) were added to a 96-well microplate in triplicate, yielding final sample concentrations ranging from 0.156 to 5.000 mg/mL (in terms of dry extract). The concentrations chosen to represent the extract levels in the NPs and equivalent concentrations of crude extracts were used in all tests for comparative purposes. Bacterial cultures grown on agar were suspended in MHB and adjusted to a 0.5 McFarland standard. A total of 75 µL of bacterial culture suspended in MHB was used as a control to ensure the broth was sterile, untreated bacterial culture (1 × 10^5^ colonies forming units (CFU)/mL) was used as a negative control, and the commercial antiseptic solution betadine (Sanofi, Sydney, New South Wales, Australia) was used as a growth inhibition control (positive control) at a final concentration of 0.1% povidone–iodine (PVP-I; *w*/*v*). The microplates were incubated for 20 h at 37 °C in a 5% CO_2_ incubator. Bacterial growth was assessed by measuring the optical density (OD) at 595 nm with the 5 s slow orbital shake using a Victor plate reader (Perkin Elmer Victor X2 Multilabel Microplate Reader, UK). The OD was used to calculate the percentage of growth inhibition of bacteria compared with the bacteria-only growth control. The MIC is determined as the lowest concentration that achieves more than 95% inhibition of bacterial growth, corresponding to the concentration at which no visible bacterial growth is observed [41]. 

The minimum bactericidal concentration (MBC) was determined as the lowest concentration that kills 99.9% of the initial bacterial population [41]. Briefly, a 10 µL aliquot from each well showing no visible bacterial growth in the MIC assay was spotted on MHA plates and incubated at 37 °C in 5% CO_2_ for 20 h. The MBCs of the extracts and AgNPs were measured by observing the viability of the initial bacterial inoculum. 

### 2.7. Statistical Analysis

The results obtained from the biological experiments were analyzed using GraphPad Software 10 (Version 10, Boston, MA, USA). All experiments were performed three times, and the values are expressed as the mean ± SD. Statistical analysis was performed using two-way analysis of variance (ANOVA), with significant differences observed at *p* < 0.05.

## 3. Results

According to the literature, *T. ferdinandiana* extracts are rich in phenolic compounds [33,42]. An analysis of both negative and positive ionizations revealed the presence of numerous statistically significant features in both the leaf and fruit extracts. The significant features were downsized to 149 metabolites upon the final filtering with an absolute fold change ≥ 2 and corrected *p* (False Discovery Rate; FDR) ≤ 0.05 against blank samples (more details are given in S1). All tentatively identified compounds are shown in Table S1.

**Figure 1:**
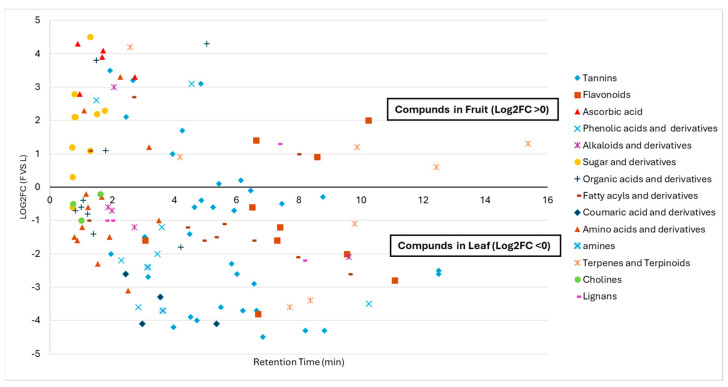
Log2FC values from liquid chromatography–mass spectroscopy (LC-MS) metabolomic profiling of *Terminalia ferdinandiana* leaf and fruit water extracts.

The identified compounds were categorized into main chemical classes, such as tannins, flavonoids, phenolic acids, ascorbic acid, alkaloids, organic acids and their derivatives, sugar and its derivatives, coumarins, and amino acids, as summarized in Figure 1. The negative and positive values of Log2FC (F vs. L) indicate that the compounds were mainly present in the leaf and fruit extracts, respectively.

### 3.1. Optimization and Characterization of AgNPs

**Figure 2:**
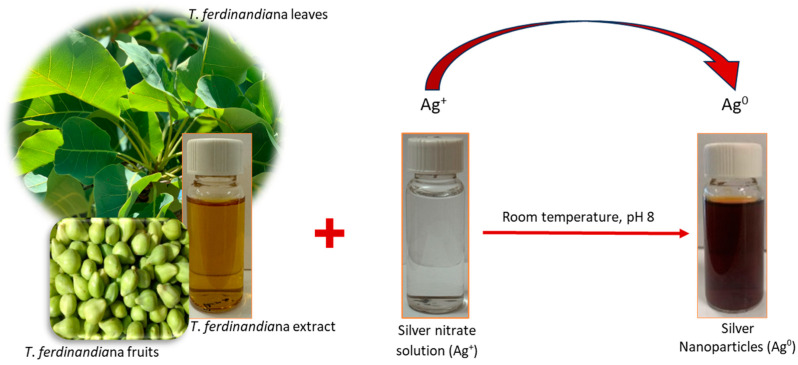
Schematic illustration of the synthesis of silver nanoparticles (AgNPs) from *T. ferdinandiana* aqueous extracts.

AgNPs of *T. ferdinandiana* extract were synthesized by mixing the AgNO_3_ aqueous solution with the extract at room temperature. The reduction of Ag^+^ to Ag^0^ was initially evident by the color change of the solution from pale yellow to reddish brown (Figure 2) with leaf extracts, whereas the solution turned brownish black with fruit extract, with no subsequent color change. This color change due to the excitation of surface plasmon vibrations in AgNPs was evaluated as primary evidence for the formation of AgNPs. 

**Figure 3:**
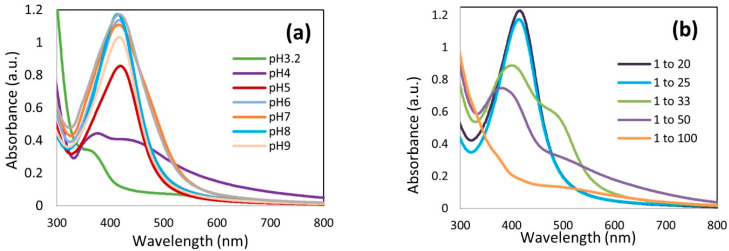
Ultraviolet–visible (UV-vis) spectra of silver (Ag) nanoparticles synthesized with leaf extract (AgKL) at (**a**) different pHs and (**b**) different ratios of Ag^+^/leaf extract at pH 8 (*w*/*w*).

**Figure 4:**
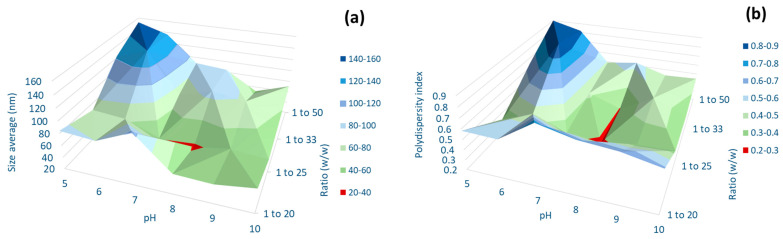
(**a**) Average particle size (nm) and (**b**) polydispersity index of AgKL at different pHs and different Ag^+^/leaf extract ratios (*w*/*w*).

The effect of the pH was assessed by adjusting the pH of the mixture from 4 up to 10. The UV-vis spectra of AgKL in Figure 3a indicate that NPs were not formed at pH 3.2 (original pH) and pH 4. From pH 5 onwards, the appearance of a single peak between 414 and 420 nm indicates the formation of AgKL. The particle size and PDI at different pH values are shown in Figure 4a,b, respectively. AgKL synthesized at pH 8 (dark red area) has a particle size between 20 and 40 nm and a PDI value between 0.2 and 0.4, which represent the most suitable conditions for the formation of stable NPs.

The results also revealed that different Ag+/*T. ferdinandiana* (*w*/*w*) ratios (1:100, 1:50, 1:33, 1:25, and 1:20) influenced the size and distribution of AgNPs. The UV-vis spectra in Figure 3b show the increasing intensity of the absorption peaks as the amount of *T. ferdinandiana* leaf extract increased, with a constant amount of Ag^+^ along with a redshift and narrow peaks. The AgKL at both 1:20 and 1:25 Ag^+^/*T. ferdinandiana* (*w*/*w*) ratios had a narrow surface plasmon band centered at approximately 415 nm at the highest intensity. However, a comparison of the PDI and particle size (area represented in dark red in Figure 4a,b) revealed that AgKL synthesized at a 1:25 Ag^+^/*T. ferdinandiana* (*w*/*w*) ratio had the smallest particle size and PDI values.

**Figure 5:**
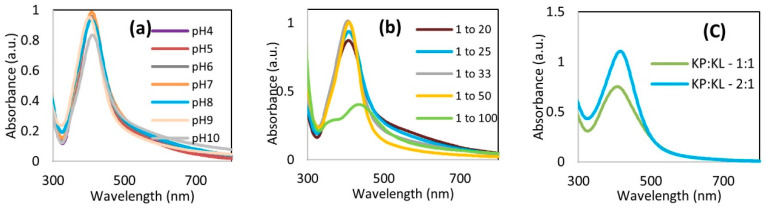
UV-vis spectra of AgKP at (**a**) different pHs and (**b**) different Ag^+^/fruit extract ratios at pH 8 (*w*/*w*) and (**c**) AgNPs synthesized with leaf and fruit extract mixture (AgKPL) at two different fruit extract/leaf extract (KP/KL) ratios at pH 8.

The assessment of the impact of pH and the Ag/*T. ferdinandiana* (*w*/*w*) ratio on the formation of AgNPs synthesized with *T. ferdinandiana* fruit extract was conducted similarly to the leaves over the range of pH 4–10 and a ratio of 1:100–1:20. The obtained surface plasmon resonance (SPR) spectra (Figure 5a,b) show similar absorption peaks with high intensities around 408 nm for fruit extract nanoparticles (AgKP) at all pH values and Ag^+^/*T. ferdinandiana* (*w*/*w*) ratios assessed. However, dynamic light scattering (DLS) results showed a larger mean particle size of AgKP, ranging from 140 to 200 nm. In addition, the NP size distribution of AgKP articulated through PDI showed a high value of >0.78. However, among all the synthesized AgKP NPs, the AgKP synthesized at pH 8 and a 1:25 ratio showed the smallest particle size and lowest PDI value, 147 nm and 0.788, respectively. 

Since AgKP has large NPs with a broad distribution, a third type of AgNP, AgKPL, was synthesized by mixing the two extracts at two different ratios (fruit/leaf, 1:1 and 2:1) under the conditions optimized for AgKP and AgKL synthesis. For NPs synthesized by combining half the amount of *T. ferdinandiana* leaf extract (KL) with fruit extract (KP; KP/KL = 2:1), the ultraviolet (UV) spectrum exhibited a narrower band with a redshift of approximately 415 nm compared with a 1:1 ratio, as shown in Figure 5c. The DLS data for AgKPL showed a 27.9 nm mean diameter with a lower PDI value (0.605) than AgKP (Table 1).

**Figure 6:**
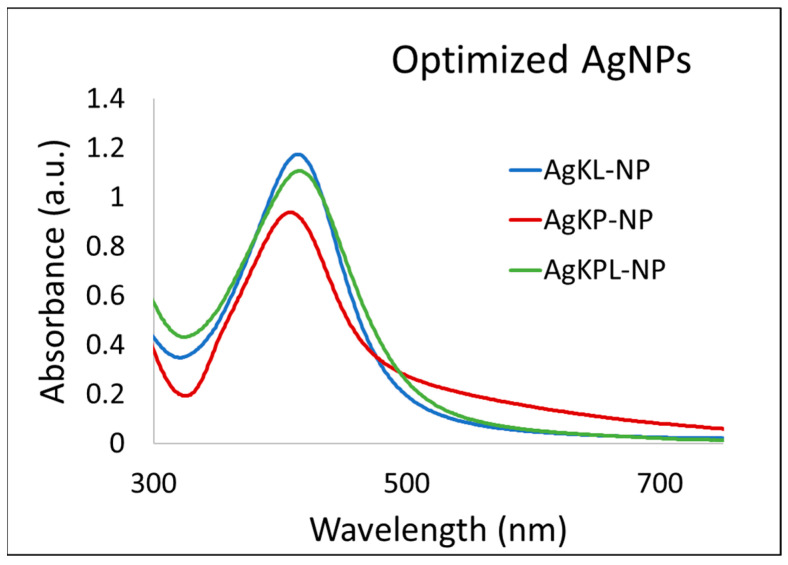
UV–vis spectra of optimized AgNPs prepared at room temperature and at pH 8.

The UV spectra of the three optimized AgNPs are compared in Figure 6. The UV-vis spectra in Figure S1 demonstrate that the characteristic peak for Ag^+^ is absent in the spectra of the AgNPs, which confirms the utilization of all Ag^+^ ions in the synthesis of NPs. 

The stability and surface charge of the AgNPs were analyzed using the zeta potential values obtained from the DLS measurements (Table 1). In our study, the synthesized AgKL, AgKP, and AgKPL have zeta potentials of −81.3 mV, −75.6 mV, and −78.6 mV, respectively, which correspond to negatively charged AgNPs. 

**Figure 7:**
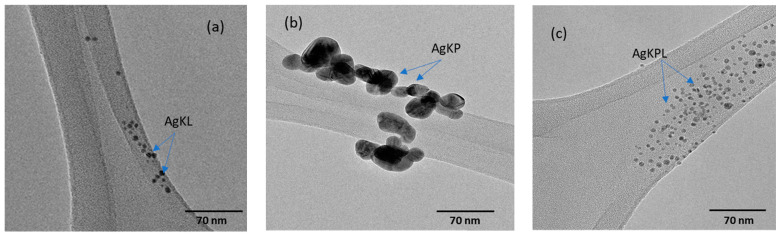
Transmission electron microscopy images of (**a**) AgKL, (**b**) AgKP, and (**c**) AgKPL synthesized from *T. ferdinandiana* extracts. AgKL: silver nanoparticles synthesized with leaf extract; AgKP: silver nanoparticles synthesized with fruit extract; AgKPL: silver nanoparticles synthesised with leaf and fruit extract mixture.

The TEM analysis revealed that the optimized AgNPs are well distributed in solution, and most of the particles have a spherical or near-spherical morphology with average diameters of 6.50 nm, 28.9 nm, and 4.40 nm for AgKL, AgKP, and AgKPL NPs, respectively, (Figure 7a–c). However, AgKP had an irregular shape, possibly due to agglomeration. Furthermore, the selected area electron diffraction pattern confirmed the crystalline structure of the AgNPs, which can be attributed to the (111), (200), (220), and (311) planes of the face-centered cubic structure of the Ag lattice of the AgNPs (Figure S2). 

The size of the AgNPs obtained using DLS was larger than that obtained using TEM. This is because DLS provides the hydrodynamic diameter of the nanoparticles, which includes the surface coating by phytochemicals or the assembly of water molecules around the nanoparticles. However, TEM provides an accurate size for the metallic part of the nanoparticles [43]. 

### 3.2. Antioxidant Activity

**Figure 8:**
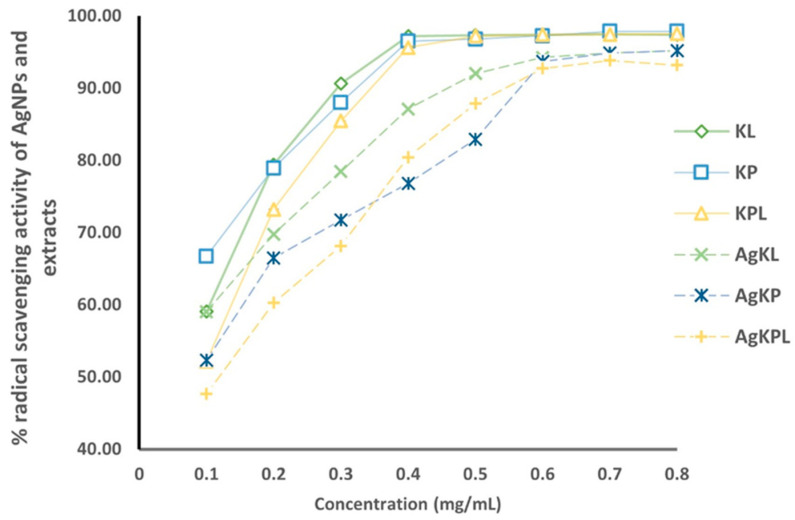
Antioxidant activities of *T. ferdinandiana* extracts and synthesized AgNPs. Results are presented as the mean ± SD. KL: *T. ferdinandiana* leaf extract; KP: *T. ferdinandiana* fruit extract; KPL: a mixture of *T. ferdinandiana* leaf and fruit extracts.

The antioxidant abilities of AgNPs and *T. ferdinandiana* extracts were assessed using DPPH, which measures their ability to convert stable purple free radicals to pale yellow. The antioxidant activities of the AgNPs and *T. ferdinandiana* extracts were examined at different concentrations (0.1–0.8 mg/mL in terms of dry extract). The radical scavenging activities of the AgNPs and *T. ferdinandiana* extracts increased with increasing concentration, as shown in Figure 8. 

All AgNPs and extracts exhibited greater than 50% free radical scavenging activity at the concentrations analyzed (Figure 8), except for AgKPL, which exhibited 47% at the lowest concentration (0.1 mg/mL). At concentrations of 0.4 mg/mL or higher, all T. ferdinandiana extracts showed more than 90% inhibition. More than 90% inhibition occurs for AgNPs at concentrations of 0.6 mg/mL or above.

### 3.3. Antibacterial Activity

The MICs and MBCs of *T. ferdinandiana* extracts and optimized AgNPs were tested for their effects on four different bacteria at different concentrations. AgKL and AgKPL exhibited antibacterial activity against both Gram-positive (*S. aureus*) and Gram-negative (*E. coli*, *P. aeruginosa*, and *P. mirabilis)* bacteria, while *T*. *ferdinandiana* extracts only showed inhibitory activity towards *S. aureus* (with an MIC of 1.25 mg/mL for KP and KPL extracts). *T. ferdinandiana* extract alone had no bactericidal effect and prevented the growth of Gram-negative bacterial strains (*E. coli*, *P. aeruginosa*, and *P. mirabilis*) at the tested concentrations (Table 2). The negative control exhibited 100% bacterial growth, indicating no inhibitory effect. The control used to confirm the sterility of the broth showed no bacterial growth. The positive control, which consisted of 0.1% povidone–iodine (*w*/*v*), demonstrated complete bacterial inhibition (100% inhibition).

*E. coli* showed higher sensitivity to AgKL and AgKPL than the other bacteria tested, and *P. aeruginosa* was relatively resistant to AgNPs. The MBC of AgKL and AgKPL against *P. aeruginosa* was 5.00 mg/mL and against *E. coli* was 0.63 mg/mL and 0.31 mg/mL, respectively (Table 2). None of the AgKP concentrations showed an antibacterial activity of more than 95% against all four microorganisms. 

## 4. Discussion

Phytochemical analysis of *T. ferdinandiana* extracts showed that the number of phytochemicals detected in the leaf extract was much higher than that in the fruit extract (Figure 1). The major compounds in the leaf extract were coumarins, flavonoids, phenolic acids, and a variety of tannins, with a remarkable diversity of tannin compounds, similar to earlier studies [36]. Additionally, loliolide, which belongs to the class monoterpenoid and is known to exhibit antioxidant, antifungal, antibacterial, and anticancer properties, ref. [44] was also seen in the leaf extract with −3.6 Log2FC (F vs. L). In contrast to the leaf extract, very few tannins and flavonoids were detected in the fruit extract, which also agrees with previous findings [33,42], where gallic acid and its derivatives were mainly found in the fruit extract. The fruit extract was rich in ascorbic acid, a promising antioxidant, similar to previous research findings [42]. The ascorbic acid content in the fruit was 16 times higher than that in the leaf extract. In addition, phenolic acids, sugar compounds, and their derivatives were identified in fruit extracts. 

Among the phenolic compounds identified, the presence of a catechol structure with hydroxyl groups on the benzene ring was responsible for the superior antioxidant properties [45]. The phenolic compounds in the leaf and fruit extracts of *T. ferdinandiana* are efficient scavengers of oxygen free radicals, owing to the hydroxyl groups on their molecular structure [45,46]. Several studies have reported the high antioxidant capacity of ascorbic acid [31,47,48,49]. Most of these phytochemicals have been previously identified in several different extracts of *T. ferdinandiana.* Specifically, previously detected compounds [50,51], including ascorbic acid, corilagin, chebulagic acid, chebulinic acid, gallic acid, quercetin, isovitexin, isoorientin/luteolin, myricetin 3-sambubioside, L-gluconolactone, proline, succinic acid, and citric acid, were identified in our study. 

Tannins are known for their bioactivity in in vivo and in vitro models and have been reported to inhibit the growth of a broad spectrum of bacterial species [52] and reduce inflammation [35]. Several tannins, including corilagin, chebulagic acid, chebulinic acid, and gallic acid, found in this work were previously reported to have antioxidant, anticancer, and antimicrobial activities [53,54,55]. These results identified ten flavonoids in both the leaf and fruit extracts of *T. ferdinandiana*. The leaf extract contained isoorientin, orientin 2″-O-gallate, isovitexin, myricetin 3-sambubioside, quercetin 3-O-(6″-acetyl-glucoside), eujambin, and pleurostimin 7-glucoside. In contrast, only two flavonoids, quercetin and orientin, were identified in the fruit extract. The presence of different flavonoids, including quercetin, vitexin, and isoorientin/luteolin, in *T. ferdinandiana* leaves has also been previously reported [50].

Plant extracts contain many reducing and stabilizing agents that result in the formation of nanoparticles of various sizes and shapes. The active chemical components can convert the metal ion into its non-ionic form, reducing it throughout the synthesis process [19,56]. As previously reported, the NP size and surface chemistry influence the absorption, biodistribution, and pharmacokinetics of products used in biomedical applications [57]. Although the process of synthesis of metallic NPs using plants is not fully understood, the polyphenolic compounds in *T. ferdinandiana* leaf and fruit extracts (presented in Table S1) can play a significant role in reducing, stabilizing, and capping Ag^+^ to Ag°, and electron donation from oxygen atoms can aid in phytochemical adsorption on AgNP surfaces [58].

One of the primary goals of our study was to produce well-stabilized homogenous NPs with small particle sizes that are suitable for antibacterial applications. Hence, we assessed the effect of the pH on AgNP synthesis, and the results of the UV spectra, PDI, and mean hydrodynamic diameter revealed that the pH of the reaction mixture had a substantial influence on both the size and dispersion of AgNPs. An NP system with a PDI value < 0.1 is considered highly monodisperse. In contrast, a PDI value > 0.4 is highly polydisperse, and a value of 0.1–0.4 indicates that the system has a moderately dispersed distribution [59].

In our study, small and stable AgNPs, between 20 and 40 nm with a PDI value between 0.2 and 0.4, were acquired at pH 8 for AgKL, indicating a moderately dispersed distribution. As shown in Figure 3a in the UV–visible spectra, at pH 8, AgKL had the highest intensity with a sharp absorption peak at 414 nm, indicating the maximum yield of uniform AgNPs. The effective synthesis of AgNPs at a higher or basic pH is in accordance with the results from previous studies [60,61,62], where the synthesis of AgNPs using *Hibiscus rosa sinensis*, *Olea europaea* leaf extract, and *Nostoc muscorum* was optimized at a basic pH (7.5–8.0). A significant influence of the reaction pH is its ability to change the electrical charges of biomolecules, which affects their capping and stabilizing abilities, which, subsequently, affect the growth of nanoparticles [62]. The alkaline environment enhanced the reducing and stabilizing capabilities of the phytochemicals in *T. ferdinandiana* leaf extract. In contrast, the degradation or inactivation of bioactive molecules at an acidic pH can be the cause for the insignificant synthesis of AgNPs [19]. The presence of larger NPs above or below the optimum pH value could be due to the uncontrolled nucleation and aggregation of AgNPs [60]. 

The results also revealed that different Ag^+^/*T. ferdinandiana* (*w*/*w*) ratios influenced the size and size distribution of AgNPs. The UV-vis spectra in Figure 3b illustrate that the peak of AgKL at a 1:25 Ag^+^/*T. ferdinandiana* (*w*/*w*) ratio has a narrow absorption peak, whereas the 1:33 and 1:50 Ag^+^/*T. ferdinandiana* (*w*/*w*) ratios showed a broader absorption peak with lower intensities, suggesting the formation of a smaller number of NPs with inhomogeneous distribution due to insufficient Ag^+^ ions. This was also reflected by the PDI and particle size values shown in Figure 4a,b, respectively. Although the UV-vis spectra showed high-intensity peaks with a 1:20 Ag^+^/*T. ferdinandiana* (*w*/*w*) ratio, the PDI and particle size were greater than 1:25. This could be attributed to the fact that increasing the initial amount of Ag ions beyond the optimum level could result in insufficient reductants and stabilizers to form stable NPs, thus encouraging the formation of agglomerates or large NPs [60,63]. 

In the case of AgKP, the mean particle size of the NPs at pH 8 and a 1:25 Ag^+^/*T. ferdinandiana* (*w*/*w*) ratio was greater than that of AgKL, ranging from 140 to 200 nm. In addition, the PDI of AgKP was much higher than that of AgKL (>0.700), indicating a highly polydisperse distribution. This was further validated by the color changes that occurred during the synthesis of AgKP, which turned brownish black rather than reddish brown in AgKL. Since the phytochemical compounds in the *T. ferdinandiana* extracts facilitate the capping and stabilizing activities of the synthesized AgNPs, it can be concluded that the high PDI and larger particle size of AgKP could be due to a lack of capping and stabilizing agents in the *T. ferdinandiana* fruit extract. 

During NP generation, reducers in plant extracts, including amino acids [64,65,66,67,68,69,70,71], lignin [72], flavonoids [73,74], and tannins [75,76], play an important role as effective capping and stabilizing agents. Moreover, terpenoids, amines, and carbonyl compounds in plant extracts facilitate the stabilization of the generated NPs by capping onto the surface of nanoparticles [77]. Ascorbic acid was found to be a good reducing agent [75] and may also act as a stabilizing agent [78]. 

The LC-MS results of this study clearly showed a higher phytochemical content in the leaf extracts than in the fruit extracts. In particular, rich capping and stabilizing agents, including amino acids [64,65,66,67,68,69,70,71], lignin [72], flavonoids [73,74], and tannins [75,76], present in leaf extracts could facilitate the synthesis of well-homogenized, stable NPs. However, the fruit extracts did not contain any of these compounds.

To provide capping and stabilizing agents, NPs were synthesized by mixing *T. ferdinandiana* leaf extract with fruit extract. This resultant AgKPL NPs showed a narrower band in the UV spectrum (indicated in Figure 5c), with a 27.9 nm mean diameter and a lower PDI value compared to AgKP. These results confirmed that the phytochemicals in the fruit extracts lack the phytochemicals required to act as capping and stabilizing agents.

The high magnitude of the zeta potential suggests strong electrostatic repellent interactions between the NPs and, thus, sufficient dispersion stability. Nanoparticles with zeta potentials higher than 20.0 mV or less than −20.0 mV have strong electrostatic repulsion and remain stable in solution [79]. The synthesized AgNPs showed highly negative values, which may prove the high stability of the synthesized NPs. These results confirm that surface coating by phytochemicals in *T. ferdinandiana* extracts provides extra stability against oxidation or agglomeration [45]. The negative surface charge of the NP suspensions indicates their high stability and low toxicity to normal cells [19,45].

The total *T. ferdinandiana* leaf and fruit extracts contain various chemicals with outstanding antioxidant activities [31,37,80,81,82]. The synthesized AgNPs were examined to determine whether they retained the antioxidant potential of *T. ferdinandiana* extracts by evaluating their DPPH free radical scavenging capacity. To elucidate the statistically significant difference in the antioxidant activity of the produced AgNPs and *T. ferdinandiana* extracts, the DPPH scavenging effects at different concentrations (0.1–0.8 mg/mL in terms of extract) were analyzed using the analysis of variance. The result indicates that the *p* value for the AgNPs and *T. ferdinandiana* extracts is less than 0.05 (*p* value > 0.001), which means that the correlation of higher DPPH antioxidant activities with the high concentration of AgNPs and *T. ferdinandiana* extracts is statistically significant. These results validated the in vitro antioxidant potential of AgNPs.

Prior studies have documented the potent antibacterial properties of *T. ferdinandiana* extracts [34,35,36] using different solvents. This study aimed to assess the antibacterial activity of aqueous *T. ferdinandiana* extracts and AgNPs. In the antibacterial efficacy assay, the negative control showed 100% bacterial growth, while the positive control demonstrated 100% growth inhibition. These results confirm that the assays are functioning correctly. The antibacterial microplate dilution test results (Table S2) showed that AgKL and AgKPL NPs displayed more than 80% inhibition against all four bacteria at the assessed concentrations, whereas the *T. ferdinandiana* extracts alone did not show a bactericidal effect (80%) at those concentrations, except for *S. aureus*. Although the extracts had inhibitory efficacy against *S. aureus*, their MIC values were greater than that of the AgNPs. KP and KPL showed only around 50% inhibition of *E. coli* and *P. mirabilis* at 2.50 mg/mL and 0.16 mg/mL concentrations, respectively. KL demonstrates around 50% inhibition against *E. coli* at 1.25 mg/mL and against *P. mirabilis* at 0.16 mg/mL (Table S2). 

These findings show that AgNPs exhibit greater antibacterial efficacy against Gram-positive and Gram-negative bacteria than *T. ferdinandiana* extracts. Although the specific mechanism of NP action is still being investigated [83,84,85], previous studies have demonstrated that AgNP activity is firmly size dependent [5,6,85]; that is, smaller NPs appear to have a greater capacity for penetrating bacteria [85,86]. These findings align with our results, as AgKP, which has a 146.6 nm average particle size, shows no inhibitory or biocidal activity against the tested bacteria because of its large particle size. Both AgKL and AgKPL NPs had smaller particle sizes of approximately 35 nm and showed good inhibitory and bactericidal effects against all tested bacteria. 

The negative charge of the AgNP surface due to phytochemical attachment enhances the electrostatic attraction between NPs and cell walls, allowing more Ag^+^ to enter the cells [87]. A previous study reported the synthesis of AgNPs from *Zizyphus spina christi* leaf extract as biocidal agents, which were found to have a stronger inhibitory effect on *S. aureus* than *E. coli.* At the same time, *P. aeruginosa* was more resistant to AgNPs than other bacteria, which agrees with our results. Variations in the cell wall compositions of Gram-positive and Gram-negative bacteria can also affect their antibacterial activities [88]. The cell walls of Gram-positive bacteria comprise a thick peptidoglycan layer composed of short peptide-cross-linked linear polysaccharide chains. This results in a stiffer structure, which makes AgNP penetration more difficult [89]. Despite the thicker cell walls of Gram-positive bacteria (*S. aureus*), AgKL and AgKPL exhibited notable toxicity against *S. aureus*. In contrast, Gram-negative bacteria have a thinner peptidoglycan layer in their cell walls [89,90]. Although AgKP demonstrated around 50% inhibition at concentrations of 1.25 mg/mL, 0.63 mg/mL, 0.63 mg/mL, and 0.15 mg/mL against *E. coli*, *S. aureus*, *P. aeruginosa*, and *P. mirabilis*, respectively (Table S2), AgKL and AgKPL showed approximately 50% inhibition of *E. coli*, *S. aureus*, and *P. mirabilis* at the lowest concentration (0.15 mg/mL) tested, whereas *P. aeruginosa* showed it at 0.31 mg/mL (Table S2).

The aqueous extract of *T. ferdinandiana* leaves formed small, well-stabilized, and homogeneous AgNPs, while fruit extract could not. However, incorporating leaf extract into the fruit extract led to the formation of small, well-structured nanoparticles, demonstrating that phytochemicals from different parts of the same plant can serve effectively as capping agents. These findings again demonstrate the difference in the presence of capping and stabilizing phytochemicals in *T. ferdinandiana* fruit extract compared with *T. ferdinandiana* leaf extract, which may directly aid in producing the desired AgNPs for antibacterial purposes.

## 5. Conclusions

To the best of our knowledge, this is the first study to introduce a novel and environmentally friendly green approach for synthesizing stable AgNPs using aqueous extracts of *T. ferdinandiana*. The formation of AgNPs was attributed to the presence of major phenolic constituents, including tannins, flavonoids, and amino acids, in the *T. ferdinandiana* extracts, as identified via UPLC-MS analysis. By optimizing the pH and Ag^+^-to-extract ratio, we achieved well-stabilized and homogenized AgNPs using *T. ferdinandiana* leaf extract and a mixture of leaf and fruit extracts at room temperature. The results indicate that the fruit extract alone was insufficient, and adding leaf extract to the reaction mixture achieved well-defined AgNPs. This demonstrates that the phytochemicals in the leaf extracts contain reducing, stabilizing, and capping agents, facilitating the synthesis of superior AgNPs. The characterization of NPs confirmed the formation of well-stabilized, spherical, and near-spherical AgNPs with a homogeneous distribution. The green-synthesized AgNPs exhibited antioxidant activity, and in vitro antibacterial testing revealed that AgKL and AgKPL have promising antibacterial effects against pathogenic microorganisms such as *E. coli*, *S. aureus*, *P. aeruginosa*, and *P. mirabilis*, outperforming AgKP. Overall, these findings suggest that AgKL and AgKPL may serve as natural and potent alternatives to antibacterial medications for pharmaceutical and biomedical applications. To maximize the potential of *T. ferdinandiana*-mediated AgNPs as nutraceuticals, further research could be conducted to enhance their bioavailability, assess drug interactions, and evaluate their therapeutic efficacy in clinical trials. 

## Figures and Tables

**Table 1:** Zeta potential, PDI value, and mean hydrodynamic diameter of optimized AgNPs.

AgNPs	Zeta Potential (Mean) (mV)	PDI	Mean Hydrodynamic Diameter (nm)
AgKL	−81.3	0.287	37.4
AgKP	−75.6	0.788	147
AgKPL	−78.6	0.605	33.4

AgKL: silver nanoparticles synthesized with leaf extract; AgKP: silver nanoparticles synthesized with fruit extract; AgKPL: silver nanoparticles synthesised with leaf and fruit extract mixture; AgNPs: silver nanoparticles PDI: polydispersity index.

**Table 2:** Minimum inhibitory concentration (MIC) and minimum bactericidal concentration (MBC) of *T. ferdinandiana*-meditated AgNP, and *T. ferdinandiana* extract against Gram-positive and Gram-negative bacteria (mg/mL) from antibacterial microplate dilution assay. The results were obtained from the mean of three replicates.

Tested Microorganisms	MIC (mg/mL)					
	AgKL	AgKP	AgKPL	KL	KP	KPL
*Escherichia coli*	0.31	-	0.16	-	-	-
*Staphylococcus aureus*	1.25	-	2.50	-	1.25	1.25
*Pseudomonas aeruginosa*	0.63	-	2.50	-	-	-
*Proteus mirabilis*	0.63	-	5.00	-	-	-
**Tested Microorganisms**	**MBC (mg/mL)**					
	**AgKL**	**AgKP**	**AgKPL**	**KL**	**KP**	**KPL**
*E. coli*	0.63	-	0.31	-	-	-
*S. aureus*	2.50	-	5.00	-	-	-
*P. aeruginosa*	5.00	-	5.00		-	-
*P. mirabilis*	2.50	-	5.00	-	-	-

KPL: leaf and fruit extract mixture.

## Data Availability

All data generated or analyzed during this study are included in the published article.

## Supplementary Materials

The following supporting information can be downloaded at: https://www.mdpi.com/article/10.3390/biom14121516/s1, S1: LC-MS-based metabolomics fingerprinting [39,91,92,93,94,95]; Table S1: LC-MS metabolomic profiling of *T. ferdinandiana* leaf and fruit water extracts in electrospray ionization (ESI) (−) and ESI (+) mode; Figure S1: UV–visible absorption of (a) silver nitrate (AgNO_3_), KL, KP, and KPL and (b) optimized AgKL, AgKP, AgKPL, and their supernatants; Figure S2: Selected area electron diffraction pattern of AgNPs synthesized from *T. ferdinandiana* extracts; Table S2: Average (Av) percentage inhibition of *Escherichia coli*, *Staphylococcus aureus*, *Pseudomonas aeruginosa*, and *Proteus mirabilis* at different concentrations (mg/mL). All values were expressed as the mean ± SD of three replicates.

## References

[1] Donaldson K., Stone V. (2004). Nanoscience fact versus fiction. Commun. ACM.

[2] Jadoun S., Arif R., Jangid N.K., Meena R.K. (2021). Green synthesis of nanoparticles using plant extracts: A review. Environ. Chem. Lett..

[3] Elshafie H.S., Osman A., El-Saber M.M., Camele I., Abbas E. (2023). Antifungal Activity of Green and Chemically Synthesized ZnO Nanoparticles against *Alternaria citri*, the Causal Agent Citrus Black Rot. Plant Pathol. J..

[4] Magdy G., Aboelkassim E., Abd Elhaleem S.M., Belal F. (2024). A comprehensive review on silver nanoparticles: Synthesis approaches, characterization techniques, and recent pharmaceutical, environmental, and antimicrobial applications. Microchem. J..

[5] Yin I.X., Zhang J., Zhao I.S., Mei M.L., Li Q., Chu C.H. (2020). The antibacterial mechanism of silver nanoparticles and its application in dentistry. Int. J. Nanomed..

[6] Menichetti A., Mavridi-Printezi A., Mordini D., Montalti M. (2023). Effect of size, shape and surface functionalization on the antibacterial activity of silver nanoparticles. J. Funct. Biomater..

[7] Morones J.R., Elechiguerra J.L., Camacho A., Holt K., Kouri J.B., Ramírez J.T., Yacaman M.J. (2005). The bactericidal effect of silver nanoparticles. Nanotechnology.

[8] Marassi V., Di Cristo L., Smith S.G.J., Ortelli S., Blosi M., Costa A.L., Reschiglian P., Volkov Y., Prina-Mello A. (2018). Silver nanoparticles as a medical device in healthcare settings: A five-step approach for candidate screening of coating agents. R. Soc. Open Sci..

[9] Krishnan P.D., Banas D., Durai R.D., Kabanov D., Hosnedlova B., Kepinska M., Fernandez C., Ruttkay-Nedecky B., Nguyen H.V., Farid A. (2020). Silver nanomaterials for wound dressing applications. Pharmaceutics.

[10] Ong W.T.J., Nyam K.L. (2022). Evaluation of silver nanoparticles in cosmeceutical and potential biosafety complications. Saudi J. Biol. Sci..

[11] Gajbhiye S., Sakharwade S. (2016). Silver Nanoparticles in Cosmetics. J. Cosmet. Dermatol. Sci. Appl..

[12] Toro A.U., Gupta V., Shukla S.K., Bansal P., ul Islam S., Hussain C.M., Shukla S.K. (2023). Chapter 10-Functional finishing of textile materials using silver-based functionalized nanoparticles: Health perspectives. Antiviral and Antimicrobial Coatings Based on Functionalized Nanomaterials.

[13] Guo Z., Wang Y., Huang J., Zhang S., Zhang R., Ye D., Cai G., Yang H., Gu S., Xu W. (2021). Multi-functional and water-resistant conductive silver nanoparticle-decorated cotton textiles with excellent joule heating performances and human motion monitoring. Cellulose.

[14] Abazari M., Badeleh S.M., Khaleghi F., Saeedi M., Haghi F. (2023). Fabrication of silver nanoparticles-deposited fabrics as a potential candidate for the development of reusable facemasks and evaluation of their performance. Sci. Rep..

[15] Bhardwaj A.K., Sundaram S., Yadav K.K., Srivastav A.L. (2021). An overview of silver nano-particles as promising materials for water disinfection. Environ. Technol. Innov..

[16] Radwan I.M., Potter P.M., Dionysiou D.D., Al-Abed S.R. (2020). Silver Nanoparticle Interactions with Surfactant-Based Household Surface Cleaners. Environ. Eng. Sci..

[17] Raveendran P., Fu J., Wallen S.L. (2003). Completely “green” synthesis and stabilization of metal nanoparticles. Journal of the American Chemical Society.

[18] Sharma V.K., Yngard R.A., Lin Y. (2009). Silver nanoparticles: Green synthesis and their antimicrobial activities. Adv. Colloid Interface Sci..

[19] Khandanlou R., Murthy V., Saranath D., Damani H. (2018). Synthesis and characterization of gold-conjugated *Backhousia citriodora* nanoparticles and their anticancer activity against MCF-7 breast and HepG2 liver cancer cell lines. J. Mater. Sci..

[20] Akter M., Sikder M.T., Rahman M.M., Ullah A.K.M.A., Hossain K.F.B., Banik S., Hosokawa T., Saito T., Kurasaki M. (2018). A systematic review on silver nanoparticles-induced cytotoxicity: Physicochemical properties and perspectives. J. Adv. Res..

[21] Singh P., Garg A., Pandit S., Mokkapati V.R.S.S., Mijakovic I. (2018). Antimicrobial Effects of Biogenic Nanoparticles. Nanomaterials.

[22] Fahim M., Shahzaib A., Nishat N., Jahan A., Bhat T.A., Inam A. (2024). Green Synthesis of Silver Nanoparticles: A Comprehensive Review of Methods, Influencing Factors, and Applications. JCIS Open.

[23] Rani N., Singh P., Kumar S., Kumar P., Bhankar V., Kumar K. (2023). Plant-mediated synthesis of nanoparticles and their applications: A review. Mater. Res. Bull..

[24] Ahmed S., Ahmad M., Swami B.L., Ikram S. (2016). A review on plants extract mediated synthesis of silver nanoparticles for antimicrobial applications: A green expertise. J. Adv. Res..

[25] Franceschinis G., Beverina M., Corleto M., Sosa A.M., Lillo C., Casará L.A., Alonso S.d.V., Maffia P., Montanari J., Tuttolomondo M.E. (2023). Green-synthesized silver nanoparticles using *Aloe maculata* extract as antibacterial agent for potential topical application. OpenNano.

[26] Singh A., Jain D., Upadhyay M.K., Khandelwal N., Verma H.N. (2010). Green synthesis of silver nanoparticles using *Argemone mexicana* leaf extract and evaluation of their antimicrobial activities. Dig. J. Nanomater. Biostructures.

[27] Abdel-Aziz M.S., Shaheen M.S., El-Nekeety A.A., Abdel-Wahhab M.A. (2014). Antioxidant and antibacterial activity of silver nanoparticles biosynthesized using *Chenopodium murale* leaf extract. J. Saudi Chem. Soc..

[28] Ahmed M.J., Murtaza G., Mehmood A., Bhatti T.M. (2015). Green synthesis of silver nanoparticles using leaves extract of *Skimmia laureola*: Characterization and antibacterial activity. Mater. Lett..

[29] Sultanbawa Y., Sultanbawa F. (2016). Australian Native Plants: Cultivation and Uses in the Health and Food Industries.

[30] Brand-Miller J.C., Cherikoff V., Lee A., Truswell A. (1982). An outstanding food source of Vitamin C. Lancet.

[31] Netzel M., Netzel G., Tian Q., Schwartz S., Konczak I. (2007). Native Australian fruits—A novel source of antioxidants for food. Innov. Food Sci. Emerg. Technol..

[32] Mohanty S., Cock I.E. (2012). The chemotherapeutic potential of *Terminalia ferdinandiana*: Phytochemistry and bioactivity. Pharmacogn. Rev..

[33] Sirdaarta J., Matthews B., Cock I.E. (2015). Kakadu plum fruit extracts inhibit growth of the bacterial triggers of rheumatoid arthritis: Identification of stilbene and tannin components. J. Funct. Foods.

[34] Cock I.E., Mohanty S. (2011). Evaluation of the antibacterial activity and toxicity of *Terminalia ferdinandia* fruit extracts. Pharmacogn. J..

[35] Courtney R., Sirdaarta J., Matthews B., Cock I.E. (2015). Tannin components and inhibitory activity of Kakadu plum leaf extracts against microbial triggers of autoimmune inflammatory diseases. Pharmacogn. J..

[36] McManus K., Wood A., Wright M.H., Matthews B., Greene A.C., Cock I.E. (2017). *Terminalia ferdinandiana* Exell. Extracts inhibit the growth of body odour-forming bacteria. Int. J. Cosmet. Sci..

[37] Akter S., Netzel M.E., Tinggi U., Osborne S.A., Fletcher M.T., Sultanbawa Y. (2019). Antioxidant rich extracts of *Terminalia ferdinandiana* inhibit the growth of foodborne bacteria. Foods.

[38] Ramadhania Z.M., Nahar J., Ahn J.C., Yang D.U., Kim J.H., Lee D.W., Kong B.M., Mathiyalagan R., Rupa E.J., Akter R. (2022). *Terminalia ferdinandiana* (kakadu plum)-mediated bio-synthesized ZnO nanoparticles for enhancement of anti-lung cancer and anti-inflammatory activities. Appl. Sci..

[39] Ali N.B., Ibrahim S.S.A., Alsherbiny M.A., Sheta E., El-Shiekh R.A., Ashour R.M., El-Gazar A.A., Ragab G.M., El-Gayed S.H., Li C.G. (2024). Gastroprotective potential of red onion (*Allium cepa* L.) peel in ethanol-induced gastric injury in rats: Involvement of Nrf2/HO-1 and HMGB-1/NF-κB trajectories. J. Ethnopharmacol..

[40] Elhabal S.F., Elwy H.M., Hassanin S., El-Rashedy A.A., Hamza A.A., Khasawneh M.A. (2022). Biosynthesis and Characterization of Gold and Copper Nanoparticles from Salvadora persica Fruit Extracts and Their Biological Properties. Int. J. Nanomed..

[41] Saki E. (2023). Synthesis of Nanoemulsions to Enhance Dermal Application of Phytochemicals Available in Australian Native Plants: Characterisation, Evaluation and Comparison of their Biomedical Activities. Ph.D. Thesis.

[42] Sirdaarta J., Matthews B., White A., Cock I.E. (2015). GC-MS and LC-MS analysis of Kakadu plum fruit extracts displaying inhibitory activity against microbial triggers of multiple sclerosis. Pharmacogn. Commun..

[43] Vemuri S.K., Banala R.R., Mukherjee S., Uppula P., Subbaiah G.P.V., Gpv S., Av G.R., Malarvilli T. (2019). Novel biosynthesized gold nanoparticles as anti-cancer agents against breast cancer: Synthesis, biological evaluation, molecular modelling studies. Mater. Sci. Eng. C.

[44] Silva J., Alves C., Martins A., Susano P., Simões M., Guedes M., Rehfeldt S., Pinteus S., Gaspar H., Rodrigues A. (2021). Loliolide, a new therapeutic option for neurological diseases? In vitro neuroprotective and anti-inflammatory activities of a monoterpenoid lactone isolated from codium tomentosum. Int. J. Mol. Sci..

[45] Khandanlou R., Murthy V., Wang H. (2020). Gold nanoparticle-assisted enhancement in bioactive properties of Australian native plant extracts, Tasmannia lanceolata and Backhousia citriodora. Mater. Sci. Eng. C.

[46] Dávalos A., Gómez-Cordovés C., Bartolomé B. (2004). Extending applicability of the oxygen radical absorbance capacity (ORAC−fluorescein) assay. J. Agric. Food Chem..

[47] Konczak I., Zabaras D., Dunstan M., Aguas P. (2010). Antioxidant capacity and phenolic compounds in commercially grown native Australian fruits. Food Chem..

[48] Padayatty S.J., Katz A., Wang Y., Eck P., Kwon O., Lee J.-H., Chen S., Corpe C., Dutta A., Dutta S.K. (2003). Vitamin C as an antioxidant: Evaluation of its role in disease prevention. J. Am. Coll. Nutr..

[49] Macan A.M., Kraljević T.G., Raić-Malić S. (2019). Therapeutic perspective of vitamin C and its derivatives. Antioxidants.

[50] Wright M.H., Sirdaarta J., Matthews B., Greene A.C., Cock I.E. (2016). Growth Inhibitory Activity of Kakadu Plum Extracts Against the Opportunistic Pathogenclostridium Perfringens: New Leads in the Prevention and Treatment of Clostridial Myonecrosis. Pharmacogn. J..

[51] Rayan P., Matthews B., McDonnell P.A., Cock I.E. (2015). *Terminalia ferdinandiana* extracts as inhibitors of *Giardia duodenalis* proliferation: A new treatment for giardiasis. Parasitol. Res..

[52] Buzzini P., Arapitsas P., Goretti M., Branda E., Turchetti B., Pinelli P., Ieri F., Romani A. (2008). Antimicrobial and antiviral activity of hydrolysable tannins. Mini Rev. Med. Chem..

[53] Li X., Deng Y., Zheng Z., Huang W., Chen L., Tong Q., Ming Y. (2018). Corilagin, a promising medicinal herbal agent. Biomed. Pharmacother..

[54] Dhingra A.K., Chopra B., Grewal A.S., Guarve K. (2022). Pharmacological properties of Chebulinic acid and related ellagitannins from nature: An emerging contemporary bioactive entity. Pharmacol. Res.-Mod. Chin. Med..

[55] Borges A., Ferreira C., Saavedra M.J., Simões M. (2013). Antibacterial activity and mode of action of ferulic and gallic acids against pathogenic bacteria. Microb. Drug Resist..

[56] Singh H., Desimone M.F., Pandya S., Jasani S., George N., Adnan M., Aldarhami A., Bazaid A.S., Alderhami S.A. (2023). Revisiting the green synthesis of nanoparticles: Uncovering influences of plant extracts as reducing agents for enhanced synthesis efficiency and its biomedical applications. Int. J. Nanomed..

[57] Zhang H., Liu G., Zeng X., Wu Y., Yang C., Mei L., Wang Z., Huang L. (2015). Fabrication of genistein-loaded biodegradable TPGS-b-PCL nanoparticles for improved therapeutic effects in cervical cancer cells. Int. J. Nanomed..

[58] Lee K.X., Shameli K., Miyake M., Kuwano N., Bt Ahmad Khairudin N.B., Bt Mohamad S.E., Yew Y.P. (2016). Green Synthesis of Gold Nanoparticles Using Aqueous Extract of *Garcinia mangostana* Fruit Peels. J. Nanomater..

[59] Ardani H.K., Imawan C., Handayani W., Djuhana D., Harmoko A., Fauzia V. (2017). Enhancement of the stability of silver nanoparticles synthesized using aqueous extract of *Diospyros discolor* Willd. leaves using polyvinyl alcohol. IOP Conf. Ser. Mater. Sci. Eng..

[60] Hamida R.S., Ali M.A., Sharif F.T., Sonbol H., Bin-Meferij M.M. (2023). Biofabrication of silver nanoparticles using *Nostoc muscorum* Lukesova 2/91: Optimization, characterization, and biological applications. Int. J. Nanomed..

[61] Philip D. (2010). Green synthesis of gold and silver nanoparticles using *Hibiscus rosa sinensis*. Phys. E Low-Dimens. Syst. Nanostruct..

[62] Khalil M.M.H., Ismail E.H., El-Baghdady K.Z., Mohamed D. (2014). Green synthesis of silver nanoparticles using olive leaf extract and its antibacterial activity. Arab. J. Chem..

[63] Htwe Y.Z.N., Chow W.S., Suda Y., Mariatti M. (2019). Effect of silver nitrate concentration on the production of silver nanoparticles by green method. Mater. Today Proc..

[64] Oćwieja M., Morga M. (2019). Electrokinetic properties of cysteine-stabilized silver nanoparticles dispersed in suspensions and deposited on solid surfaces in the form of monolayers. Electrochim. Acta.

[65] Figat A.M., Bartosewicz B., Liszewska M., Budner B., Norek M., Jankiewicz B.J. (2023). α-Amino Acids as Reducing and Capping Agents in Gold Nanoparticles Synthesis Using the Turkevich Method. Langmuir.

[66] Nayak N.C., Shin K. (2006). Synthesis of L-phenylalanine stabilized gold nanoparticles and their thermal stability. J. Nanosci. Nanotechnol..

[67] Akbarzadeh A., Zare D., Farhangi A., Mehrabi M.R., Norouzian D., Tangestaninejad S., Moghadam M., Bararpour N. (2009). Synthesis and characterization of gold nanoparticles by tryptophane. Am. J. Appl. Sci..

[68] Laban B., Ralević U., Petrović S., Leskovac A., Vasić-Anićijević D., Marković M., Vasić V. (2020). Green synthesis and characterization of nontoxic L-methionine capped silver and gold nanoparticles. J. Inorg. Biochem..

[69] Mu X., Qi L., Dong P., Qiao J., Hou J., Nie Z., Ma H. (2013). Facile one-pot synthesis of L-proline-stabilized fluorescent gold nanoclusters and its application as sensing probes for serum iron. Biosens. Bioelectron..

[70] Shankar S., Rhim J.-W. (2015). Amino acid mediated synthesis of silver nanoparticles and preparation of antimicrobial agar/silver nanoparticles composite films. Carbohydr. Polym..

[71] Shumi G., Demissie T.B., Eswaramoorthy R., Bogale R.F., Kenasa G., Desalegn T. (2023). Biosynthesis of silver nanoparticles functionalized with histidine and phenylalanine amino acids for potential antioxidant and antibacterial activities. ACS Omega.

[72] Ito N.M., de Andrade Mendes Filho A., dos Santos D.J., dos Santos L.T. (2024). Synthesis of silver nanoparticles using modified lignin as a reducing agent. Next Mater..

[73] Jain S., Mehata M.S. (2017). Medicinal plant leaf extract and pure flavonoid mediated green synthesis of silver nanoparticles and their enhanced antibacterial property. Sci. Rep..

[74] Sathishkumar P., Gu F.L., Zhan Q., Palvannan T., Mohd Yusoff A.R. (2018). Flavonoids mediated ‘Green’ nanomaterials: A novel nanomedicine system to treat various diseases–Current trends and future perspective. Mater. Lett..

[75] Gibała A., Żeliszewska P., Gosiewski T., Krawczyk A., Duraczyńska D., Szaleniec J., Szaleniec M., Oćwieja M. (2021). Antibacterial and antifungal properties of silver nanoparticles-effect of a surface-stabilizing agent. Biomolecules.

[76] Barnaby S.N., Yu S.M., Fath K.R., Tsiola A., Khalpari O., Banerjee I.A. (2011). Ellagic acid promoted biomimetic synthesis of shape-controlled silver nanochains. Nanotechnology.

[77] Liu L., Yu C., Ahmad S., Ri C., Tang J. (2023). Preferential role of distinct phytochemicals in biosynthesis and antibacterial activity of silver nanoparticles. J. Environ. Manag..

[78] Sood A., Arora V., Shah J., Kotnala R.K., Jain T.K. (2016). Ascorbic acid-mediated synthesis and characterisation of iron oxide/gold core–shell nanoparticles. J. Exp. Nanosci..

[79] Karakoçak B.B., Raliya R., Davis J.T., Chavalmane S., Wang W.-N., Ravi N., Biswas P. (2016). Biocompatibility of gold nanoparticles in retinal pigment epithelial cell line. Toxicol. Vitr..

[80] Konczak I., Maillot F., Dalar A. (2014). Phytochemical divergence in 45 accessions of *Terminalia ferdinandiana* (Kakadu plum). Food Chem..

[81] Tan A.C., Konczak I., Ramzan I., Zabaras D., Sze D.M.-Y. (2011). Potential antioxidant, antiinflammatory, and proapoptotic anticancer activities of Kakadu Plum and illawarra plum polyphenolic fractions. Nutr. Cancer.

[82] Konczak I., Zabaras D., Dunstan M., Aguas P. (2010). Antioxidant capacity and hydrophilic phytochemicals in commercially grown native Australian fruits. Food Chem..

[83] Pal S., Tak Y.K., Song J.M. (2007). Does the antibacterial activity of silver nanoparticles depend on the shape of the nanoparticle? A study of the Gram-negative bacterium *Escherichia coli*. Appl. Environ. Microbiol..

[84] Sondi I., Salopek-Sondi B. (2004). Silver nanoparticles as antimicrobial agent: A case study on *E. coli* as a model for gram-negative bacteria. J. Colloid Interface Sci..

[85] Bruna T., Maldonado-Bravo F., Jara P., Caro N. (2021). Silver nanoparticles and their antibacterial applications. Int. J. Mol. Sci..

[86] Franci G., Falanga A., Galdiero S., Palomba L., Rai M., Morelli G., Galdiero M. (2015). Silver nanoparticles as potential antibacterial agents. Molecules.

[87] Abbaszadegan A., Ghahramani Y., Gholami A., Hemmateenejad B., Dorostkar S., Nabavizadeh M., Sharghi H. (2015). The effect of charge at the surface of silver nanoparticles on antimicrobial activity against gram-positive and gram-negative bacteria: A preliminary study. J. Nanomater..

[88] Dakal T.C., Kumar A., Majumdar R.S., Yadav V. (2016). Mechanistic basis of antimicrobial actions of silver nanoparticles. Front. Microbiol.

[89] Rautela A., Rani J., Debnath M. (2019). Green synthesis of silver nanoparticles from *Tectona grandis* seeds extract: Characterization and mechanism of antimicrobial action on different microorganisms. J. Anal. Sci. Technol..

[90] Silhavy T.J., Kahne D., Walker S. (2010). The bacterial cell envelope. Cold Spring Harb. Perspect. Biol..

[91] Fraisier-Vannier O., Chervin J., Cabanac G., Puech V., Fournier S., Durand V., Amiel A., André O., Benamar O.A., Dumas B. (2020). MS-CleanR: A Feature-Filtering Workflow for Untargeted LC–MS Based Metabolomics. Anal. Chem..

[92] Tsugawa H., Kind T., Nakabayashi R., Yukihira D., Tanaka W., Cajka T., Saito K., Fiehn O., Arita M. (2016). Hydrogen Rearrangement Rules: Computational MS/MS Fragmentation and Structure Elucidation Using MS-FINDER Software. Anal. Chem..

[93] Alsherbiny M.A., Bhuyan D.J., Radwan I., Chang D., Li C.G. (2021). Metabolomic identification of anticancer metabolites of Australian propolis and proteomic elucidation of its synergistic mechanisms with doxorubicin in the MCF7 cells. Int. J. Mol. Sci..

[94] Ogaly H.A., Alsherbiny M.A., El Badawy S.A., Abd-Elsalam R.M., Li C.G., Azouz A.A. (2021). Gastroprotective effects and metabolomic profiling of Chasteberry fruits against indomethacin-induced gastric injury in rats. J. Funct. Foods.

[95] Dührkop K., Fleischauer M., Ludwig M., Aksenov A.A., Melnik A.V., Meusel M., Dorrestein P.C., Rousu J., Böcker S. (2019). SIRIUS 4: A rapid tool for turning tandem mass spectra into metabolite structure information. Nat. Methods.

