# Supplementary material for: Green Synthesis of Terminalia ferdinandiana Exell-Mediated Silver Nanoparticles and Evaluation of Antibacterial Performance
Source: Biomolecules. 2024 Nov 27;14(12):1516. doi: 10.3390/biom14121516 (PMC11673889; doi:10.3390/biom14121516)
Supplement: Supplementary file 1 [file biomolecules-14-01516-s001.zip › biomolecules-3253679-supplementary.pdf]

Article

Green Synthesis of *Terminalia ferdinandiana* Exell-Mediated Silver Nanoparticles and Evaluation of Antibacterial Performance

W. Hansi S. Alwis [1], Vinuthaa Murthy [1,*], Hao Wang [1], Roshanak Khandanlou [1] and Pappu Kumar Mandal [2]

1 Faculty of Science and Technology, Charles Darwin University, Darwin, NT 0810, Australia; hansi.alwis@cdu.edu.au (W.H.S.A.); hao.wang@cdu.edu.au (H.W.); roshanak_bch@yahoo.com (R.K.)

2 Menzies School of Health Research, Darwin, NT 0810, Australia; pappukmandal@gmail.com

* Correspondence: vinuthaa.murthy@cdu.edu.au; Tel.: +61-889466794

S1. LCMS- based Metabolomics fingerprinting

The extracts were dissolved in water at a concentration of 5 mg mL^{-1} and filtered through a 0.2 µm PTFE filter for further UPLC-MS analyses. LC–MS/MS analysis was performed using an Agilent 1290 HPLC system interfaced with an Agilent 6546 quadrupole time of flight mass spectrometer with dual AJS electrospray ion source (ESI) as reported in literature [1]. Agilent MassHunter Data Acquisition was used for data acquisition Version 11.0 build 11.022101. The samples were analysed in both positive and negative modes for MS1 acquisition along with pooled quality control sample acquired 5 times in iterative AutoMSMS; Data dependant acquisition mode (DDA). In MS1 sample were scanned from 50-1200 m/z at 3 spectra per second and 0 V CE; where in DDA iterative scans of the pooled samples; a narrow isolation width MS/MS of ~1.3 amu, 5 maximum precursors per cycle with activated active exclusion after 2 spectrum for 0.1 min and iterative mass error of 10 ppm and 0.1 min exclusion tolerance. MS and MSMS range of 50-1700 m/z were implemented with fixed collision energies formula of (slope *m/z/100+offset) with slope of 3.5 and offset of 10 v. Gas and sheath gas temperature was set at 200 and 300 °C, respectively with a 12 L/min gas flow, and 35 L/min sheath gas flow, together with 3000 or 3500 V for capillary voltage in negative and positive modes. Other source parameters such as the fragmentor, skimmer1 and octopoleRFPeak were set to 122, 45 and 750 respectively with 0 V nozzle voltage. Agilent TOF reference mass solution kit (G1969-85001) was simultaneously infused to calibrate masses.

A 0.4 ml/min flow rate was implemented. Water and 90% acetonitrile were used as mobile phases A and B, respectively with 0.1 % formic acid and 10 mM ammonium formate. The following gradient was used; 1% B inclined to 40-70% B at 11-13 min then inclined to 100% B at 15 min and kept for 1min then declined to 1 % B at 17 min followed by 2 min conditioning. ACQUITY UPLC HSS-T3 Column (1.8 µm, 2.1 X 100 mm, Waters Corporation, Milford, USA) with a 2.1 X 5mm T3 VanGurd™ PreColumn (Waters Corporation, Milford, USA) was used, and the column temperature was kept at 45 °C.

Raw data were processed with MSDIAL version 4.92 where negative and positive mode batched were processed separately using 0.01 and 0.025 Da for MS1 and MS2 tolerances, 0.1 Da mass slice width, 1000 amplitude minimum peak height and aligned to a pooled quality control reference (QC) with 0.05 min RT tolerance and 0.015 Da MS1 tolerance. The Riken public spectral library version

S17 was initially implemented during MSDIAL processing for metabolites identification. MSDIAL-exported features were further cleaned and processed using MS-CleanR with minimum blank ratio set to 0.8 and a maximum relative standard deviation (RSD) set to 100 [2]. The maximum mass difference for feature relationship detection was set to 0.005 Da, and the maximum RT difference was set to 0.025 min. The Pearson correlation links were applied for a correlation ≥ 0.8 and a p-value significance threshold = 0.05. Two peaks were kept in each cluster for further database search, including the most connected and the most abundant feature in the cluster. The MSCleanR-retained features were annotated with MS-FINDER version 3.52 [3]. The MS1 and MS2 tolerances were set to 5 and 15 ppm, respectively with a 1% relative abundance cut-off. The formula finder was processed with C, H, O, P, S and N atoms with 20% isotopic ratio tolerance. Filtered compounds were mined by MSFinder based on exact mass and fragmentation using generic databases included in MS-FINDER (i.e., UNPD, COCONUT, HMDB, FooDB, CHEBI and LipidMaps). Feature filtration strategies were furtherly adopted to retain the major and differential metabolites [1,4,5]. The statistically significant features with absolute fold change ≥ 2 and corrected P (FDR) ≤ 0.05 against blank samples were kept. Agilent Mass profiler professional version 15.1 was used to filter blank, ANOVA testing and fold change calculation The annotation of the selected features were manually checked using Sirius [6]

Table S1. LCMS metabolomic profiling of *T. ferdinandiana* leaf and fruit water extracts in ESI (−) and ESI (+) mode

RT min	m/z	Adduct	Formula	Tentative ID	Class	Log2FC (F vs L)
**0.714**	195.051	[M-H]-	C6H12O7	D-Gluconic acid	Sugar acids and derivatives	1.2
**0.722**	165.040	[M-H]-	C5H10O6	Arabinonic acid	Sugar acids and derivatives	0.3
**0.734**	215.033	[M+Cl]-	C6H12O6	Inositol	Sugar alcohol and derivatives	-0.6
**0.751**	104.108	[M]+	C5H13NO	Choline	Cholines	-0.5
**0.786**	116.071	[M+H]+	C5H9NO2	Proline	Amino acids	-1.5
**0.792**	177.040	[M-H]-	C6H10O6	L-Gluconolactone	Gamma butyrolactones	2.1
**0.798**	387.112	[M+CHO2]-	C12H22O11	Sucrose	Disaccharides	2.8
**0.818**	133.015	[M-H]-	C4H6O5	Malic acid	Beta hydroxy acids and derivatives	-0.7
**0.826**	360.150	[M+NH4]+	C12H22O11	Trehalose	Disaccharides	2.1
**0.851**	300.107	[M+K]+	C9H19N5O4	Arginyl-Serine	Dipeptides	-2.4
**0.907**	175.026	[M-H]-	C6H8O6	Vitamin C;ascorbic acid	Butenolides	4.3
**0.962**	177.039	[M+H]+	C6H8O6	Vitamin C;ascorbic acid*	Butenolides	2.8

**1.003**	191.020	[M-H]-	C6H8O7	Citric acid	Tricarboxylic acids and derivatives	-0.6
**1.014**	104.107	[M]+	C5H13NO	Choline*	Cholines	-1
**1.047**	130.087	[M+H]+	C6H11NO2	DL-Pipecolinic acid	Alpha amino acids	-1.2
**1.076**	133.014	[M-H]-	C4H6O5	Malic acid*	Beta hydroxy acids and derivatives	-0.4
**1.108**	234.133	[M+H]+	C10H19NO5	Mevalonic-GABA	Amino acids and derivatives	2.3
**1.161**	146.092	[M+H]+	C5H11N3O2	4-Guanidinobutanoic acid	Gamma amino acids and derivatives	-0.2
**1.213**	129.019	[M-H]-	C5H6O4	Itaconic acid	Fatty acyls	-1
**1.22**	205.035	[M-H]-	C7H10O7	2-Methylcitric acid	Tricarboxylic acids and derivatives	-0.8
**1.224**	229.154	[M+H]+	C11H20N2O3	2-amino-4-methylpentanoyl pyrrolidine-2-carboxylate	Amino acids and derivatives	-0.6
**1.267**	147.029	[M-H]-	C5H8O5	D-(-)-Citramalic acid	Fatty acyls	1.1

**1.296**	500.102	[2M+ACN+H]+	C5H12NO7P	5-Phosphoribosylamine	Pentose phosphates	1.1
**1.299**	351.056	[M-H]-	C12H16O12	a-L-threo-4-Hex-4-enopyranuronosyl-D-galacturonic acid	Sugar acids and derivatives	4.5
**1.396**	117.019	[M-H]-	C4H6O4	Succinic acid	Dicarboxylic acids and derivatives	-1.4
**1.503**	615.059	[2M+K]+	C12H16O4S2	Malotilate	Dicarboxylic acids and derivatives	3.8
**1.504**	599.085	[2M+Na]+	C12H16O4S2	Malotilate*	Dicarboxylic acids and derivatives	3.8
**1.508**	404.118	[M+ACN+Na]+	C12H20O11	3'-Ketolactose	Disaccharides	2.2
**1.547**	132.102	[M+H]+	C6H13NO2	Isoleucine	Alpha amino acids	-2.3
**1.623**	104.107	[M]+	C5H13NO	Choline*	Cholines	-0.2
**1.677**	116.070	[M+H]+	C5H9NO2	Proline*	Amino acids	-0.3
**1.691**	175.025	[M-H]-	C6H8O6	D-(-)-Isoascorbic acid	Butenolides	3.9

**1.719**	351.056	[M-H]-	C6H8O6	D-(-)-Isoascorbic acid*	Butenolides	4.1
**1.77**	234.133	[M+ACN+H]+	C8H16O5	3,4-di-O-methyl-rhamnose	Hexoses	2.3
**1.792**	229.154	[M+H]+	C11H20N2O3	Isoleucylproline	Dipeptides	-0.3
**1.792**	191.019	[M-H]-	C6H8O7	Citric acid	Organic acids	1.1
**1.795**	439.171	[M+Na]+	C23H28O7	Virgatusin	7,7' epoxylignans	-1
**1.857**	332.134	[M+H]+	C14H21NO8	5'-O-beta-D-Glucosylpyridoxine	Carbohydrate and conjugates	-2.2
**1.875**	123.055	[M+H]+	C6H6N2O	Niacinamide	Pyridines and derivatives	-0.6
**1.916**	228.086	[M+H]+	C10H13NO5	L-Arogenic acid	L-alpha-amino acids	-1.5
**1.937**	243.050	[M-H]-	C10H12O7	1-O-Galloylglycerol	Galloyl esters	3.5
**1.96**	331.067	[M-H]-	C13H16O10	beta-Glucogallin	Tannins	-2
**1.977**	439.171	[M+Na]+	C23H28O7	Epimagnolin	Furanoid lignans	-1
**2.002**	296.133	[M+H]+	C12H17N5O4	N-6-(2-Hydroxyethyl)-Adenosine	Purine nucleosides	-0.7
**2.05**	229.154	[M+H]+	C11H20N2O3	Leucylproline	Dipeptides	-0.4
**2.06**	182.045	[M-H]-	C8H9NO4	4-Pyridoxic acid	Pyridines and derivatives	3
**2.269**	318.081	[M+Na]+	C10H17NO9	(S)-malyl alpha-D-glucosaminide	Alpha amino acids and derivatives	3.3

**2.301**	213.004	[M-H]-	C8H6O7	3,4,6-trihydroxybenzene-1,2-dicarboxylic acid	Hydrozybenzoic acid derivatives	-2.2
**2.423**	314.091	[M+H]+	C11H15N5O4S	Methylthioadenosine Sulfoxide	Nucleoside and nucleotide analogues	-4
**2.443**	281.031	[M-H]-	C12H10O8	2-O-Caffeoyltartronic acid	Coumaric acids and derivatives	-2.6
**2.456**	331.066	[M-H]-	C13H16O10	beta-Glucogallin	Galloyl esters	2.1
**2.5**	225.003	[M-H]-	C10H10O2S2	3,3'-Dithiobis[2-methylfuran]	Heteroaromatic compounds	-0.5
**2.515**	166.087	[M+H]+	C9H11NO2	Phenylalanine	Amino acids and derivatives	-3.1
**2.578**	527.160	[M-H]-	C24H32O11S	17-beta-estradiol 3-sulfate-17-(beta-D-glucuronide)	Steroidal glycosides	4.2
**2.652**	131.035	[M-H]-	C5H8O4	Ethylmalonic acid	Fatty acyls	2.7
**2.68**	301.056	[M-H]-	C12H14O9	5-Galloyloxy-3,4-dihydroxypentanoic acid	Galloyl esters	3.2
**2.723**	328.139	[M+H]+	C15H21NO7	Sesbanimide A	Piperidinediones	-1.2
**2.735**	175.024	[M-H]-	C6H8O6	D-(-)-Isoascorbic acid*	Butenolides	3.3

**2.85**	281.030	[M-H]-	C12H10O8	2,5,6,8-tetrahydroxy-3,7-dimethoxy-1,4-dihydronaphthalene-1,4-dione	Phenolic acids	-3.6
**2.897**	289.091	[M+H]+	C12H16O8	Glucosylisomaltol	O-glycosyl compounds	0.1
**2.971**	291.014	[M-H]-	C13H8O8	Phyllanthusiin E	7,8-dihydroxycoumarins	-4.1
**2.991**	323.004	[M+K-2H]-	C7H15N2O8P	Glycineamideribotide	Glycinamide ribonucleotides	-3.3
**2.999**	301.056	[M-H]-	C12H14O9	Pyrogallol-2-O-glucuronide	Phenolic glycosides	-0.8
**3.039**	220.118	[M+H]+	C9H17NO5	Pantothenic acid	Secondary alcohols	0.3
**3.058**	545.114	[M-H]-	C29H22O11	[2-[3-(3,4,5-trihydroxybenzoyl)oxyphenyl]-3,4-dihydro-2H-chromen-3-yl] 3,4,5-trihydroxybenzoate	Gallic acid and derivatives	-1.5
**3.073**	611.124	[M-H]-	C26H28O17	Myricetin 3-Sambubioside	Flavonoid glycoside	-1.6
**3.149**	206.139	[M+H]+	C9H19NO4	Pantothenol	N-acyl amines	-2.4
**3.153**	783.067	[M-H]-	C34H24O22	5'-Desgalloylstachyurin	Hydrolyzable tannins	-2.7
**3.192**	180.101	[M+H]+	C10H13NO2	Fusaric acid	Amino fatty acids	1.2
**3.306**	165.056	[M-H]-	C9H10O3	Phloretic Acid	Phenylpropanoic acid	0.1

**3.459**	285.061	[M-H]-	C12H14O8	Uralenneoside	p-Hydroxybenzoic acid alkyl esters	-2
**3.501**	205.097	[M+H]+	C11H12N2O2	Tryptophan	Indolyl carboxylic acids and derivatives	-1
**3.546**	307.01	[M-H]-	C13H8O9	3-(5-Acetyl-2-carboxyfuran-3-yl)furan-2,5-dicarboxylic acid	Coumarin and derivatives	-3.3
**3.634**	122.096	[M+H]+	C8H11N	Phenylethylamine	Phenethylamines	-3.7
**3.942**	483.078	[M-H]-	C20H20O14	1,6-Digalloyl-beta-D-glucopyranose	Tannins	1
**3.983**	451.051	[M-H]-	C19H16O13	3,4-Hexahydroxydiphenoylarabinose	Hydrolyzable tannins	-4.2
**4.18**	383.131	[M+Na]+	C16H24O9	Deoxyloganic acid	Iridoid O-glycosides	0.9
**4.266**	483.079	[M-H]-	C20H20O14	1,6-Digalloyl-beta-D-glucopyranose*	Tannins	1.7
**4.38**	293.124	[M-H]-	C12H22O8	Ethyl (S)-3-hydroxybutyrate glucoside	Fatty acyl glycosides of mono- and disaccharides	-1.2
**4.498**	483.07	[M-H]-	C20H20O14	1,6-Digalloyl-beta-D-glucopyranose*	Tannins	-1.4
**4.523**	433.040	[M-H]-	C19H14O12	Ellagic acid arabinoside	Hydrolyzable tannins	-3.9

**4.564**	183.029	[M-H]-	C8H8O5	3,4-dihydroxy-5-methoxybenzoic acid	Hydroxy benzoic acid and derivatives	3.1
**4.733**	433.041	[M-H]-	C19H14O12	Ellagic acid arabinoside	Hydrolyzable tannins	-4
**4.854**	169.014	[M-H]-	C7H6O5	Gallic acid	Gallic acids and dervatives	3.1
**4.924**	401.181	[M+H]+	C19H28O9	Corchoionoside B	Fatty acyl glycosides of mono- and disaccharides	-1.6
**5.044**	305.071	[M-H]-	C12H18O7S	(3R,7S)-12-Hso4-Ja	Jasmonic acids	-0.7
**5.045**	95.0127	[M+H-H2O]+	C5H4O3	2-Furoic acid	Furoic acids	4.3
**5.245**	785.083	[M-H]-	C34H26O22	Heterophylliin A	Hydrolyzable tannins	-0.6
**5.317**	406.207	[M+NH4]+	C18H28O9	7-Epi-12-hydroxyjasmonic acid glucoside	Fatty acyl glycosides of mono- and disaccharides	-1.5
**5.354**	273.007	[M-H]-	C10H10O7S	Ferulic acid 4-O-sulfate	Coumaric acid and derviatives	-4.1
**5.43**	633.074	[M-H]-	C27H22O18	Corilagin	Hydrolyzable tannins	0.1
**5.504**	969.083	[M-H]-	C41H30O28	Phyllanthusiin B	Hydrolyzable tannins	-3.6
**5.611**	371.098	[M-H]-	C16H20O10	Dihydroferulic acid 4-O-glucuronide	Phenolic glycosides	-0.2

**5.849**	6350906	[M-H]-	C27H24O18	1,3,6-Trigalloylglucose	Tannins	-2.3
**5.92**	477.103	[M-H]-	C22H22O12	coumaroyl(-6)L-Glc(b)-O-galloyl	Tannins	-0.7
**6.024**	969.083	[M-H]-	C41H30O28	Valolaginic acid	Hydrolyzable tannins	-2.6
**6.205**	925.093	[M-H]-	C40H30O26	Pelargoniin A	Hydrolyzable tannins	-3.7
**6.451**	951.074	[M-H]-	C41H28O27	Sanguiin H11	Hydrolyzable tannins	-0.1
**6.508**	449.109	[M+H]+	C21H20O11	Isoorientin	Flavonoid glycosides	-0.6
**6.568**	953.089	[M-H]-	C41H30O27	Chebulagic acid	Hydrolyzable tannins	-2.9
**6.623**	483.069	[M+Cl]-	C21H20O11	Orientin	Flavonoid glycosides	1.4
**6.644**	937.093	[M-H]-	C41H30O26	Nupharin A	Hydrolyzable tannins	-3.7
**6.697**	493.098	[M-H]-	C22H22O13	Pleurostimin 7-Glucoside	Flavonoid glycosides	-3.8
**6.854**	787.099	[M-H]-	C34H28O22	1,2,4,6-Tetragalloyl-beta-D-glucopyranose	Tannins	-4.5
**7.301**	431.098	[M-H]-	C21H20O10	Isovitexin	Flavonoid glycosides	-1.6
**7.358**	545.198	[M+Na]+	C26H34O11	lariciresinol-glucoside	Lignan glycosides	1.3
**7.394**	599.104	[M-H]-	C28H24O15	Orientin 2''-O-Gallate	Flavonoid glycosides	-1.2
**7.464**	955.105	[M-H]-	C41H32O27	Chebulinic acid	Hydrolyzable tannins	-0.5
**7.719**	197.117	[M+H]+	C11H16O3	Loliolide	Benzofurans	-3.6

**7.937**	520.275	[M+NH4]+	C24H38O11	Eriojaposide A	Fatty acyl glycosides of mono- and disaccharides	-2.1
**8.152**	540.244	[M+NH4]+	C26H34O11	Urolignoside	Lignan glycosides	-2.2
**8.594**	395.204	[M+Na]+	C23H22O6	rotenone	Rotenones	0.9
**8.787**	461.071	[M-H]-	C21H18O12	3-O-Methylducheside A	Hydrolyzable tannins	-0.3
**8.832**	315.014	[M-H]-	C15H8O8	3-O-Methylellagic acid	Hydrolyzable tannins	-4.3
**9.553**	524.140	[M+NH4]+	C23H22O13	Quercetin 3-O-(6''-acetyl-glucoside)	Flavonoid-3-O-glycosides	-2
**9.612**	659.194	[M+FA-H]-	C38H30O8	Spiro-Oxanthromicin A	Phenanthrenes and derivatives	-2.1
**9.795**	557.199	[M+Na]+	C27H34O11	Phillyrin	Terpene lactone	-1.1
**9.881**	689.387	[M+Na]+	C36H58O11	Sericoside	Triterpenoids	1.2
**10.23**	301.035	[M-H]-	C15H10O7	Quercetin	Flavonoid aglycone	2
**10.25**	471.128	[M-H]-	C24H24O10	1,6-Bis-O-(4-Hydroxycinnamoyl)Glucose	Hydroxycinnamic acid and derivatives	-3.5
**11.08**	657.108	[M-H]-	C30H26O17	Eujambin	Flavonoid-3-O-glycosides	-2.8
**12.41**	673.392	[M+Na]+	C36H58O10	Lucyoside N	Triterpene saponins	0.6

**15.37**	532.384	[M+H]+	$C_{28}H_{53}NO_8$	Thermolide E	Diterpene lactones	1.3

Figure S1

The supernatant of extracts and AgNPs was obtained by centrifuging at 14000 rpm for 30 min.

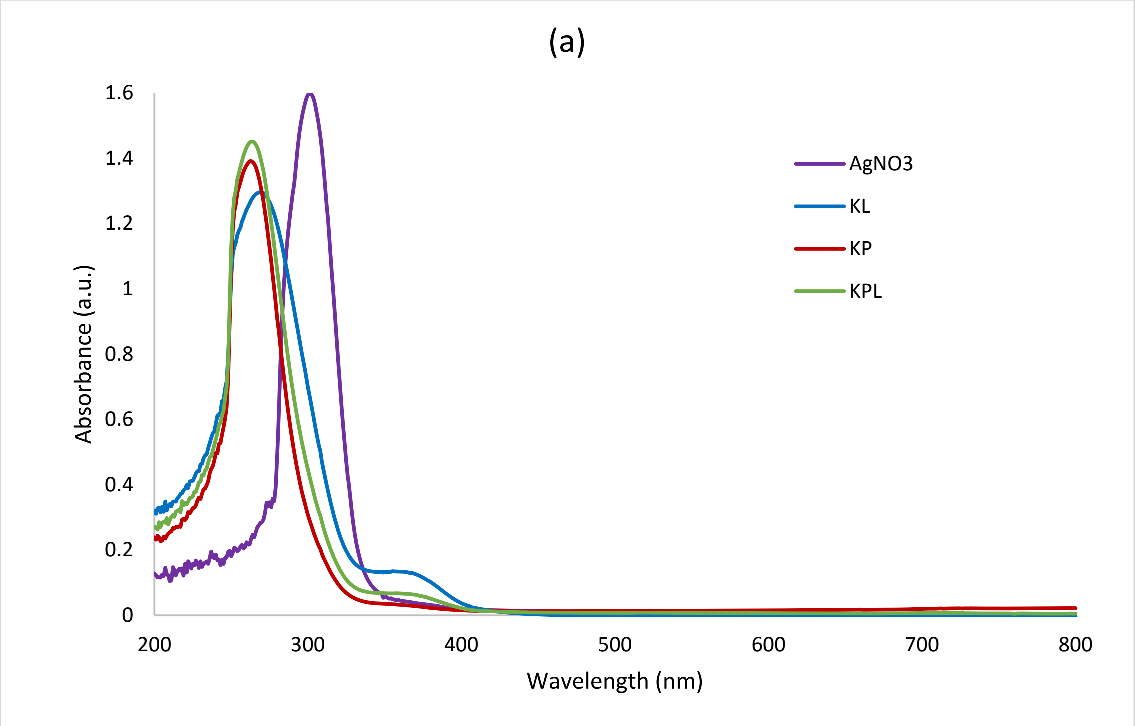

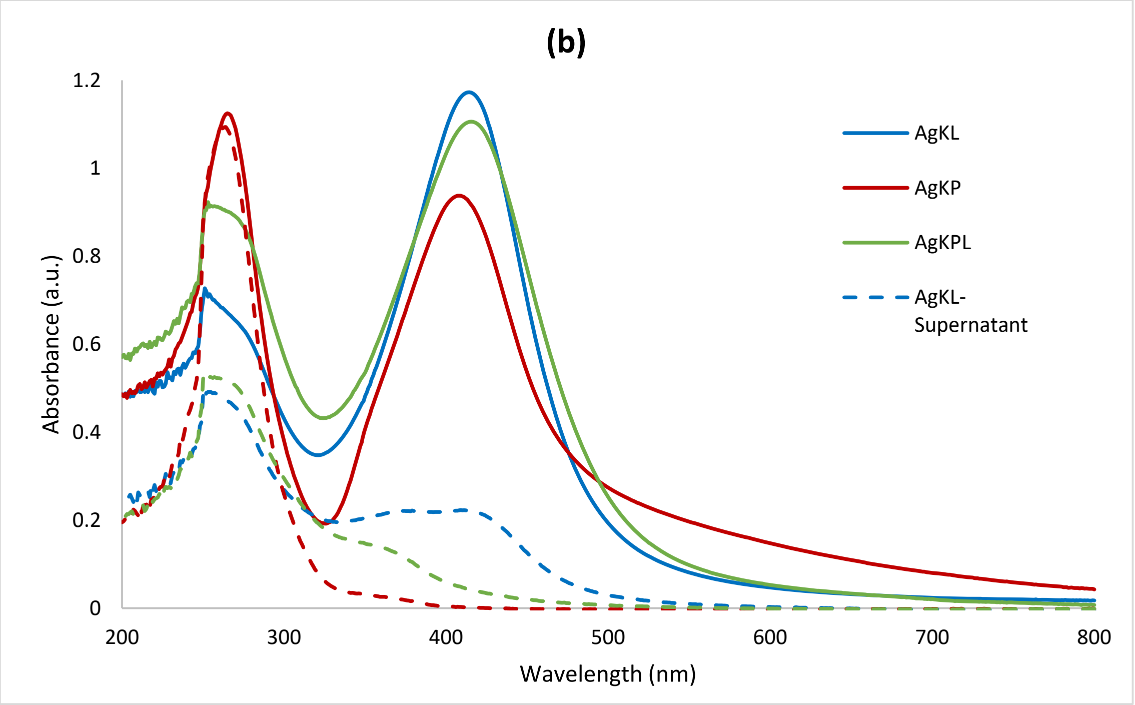

Figure S1. UV-visible absorption of (a) $AgNO_3$, KL, KP and KPL (b) optimized AgKL, AgKP, AgKPL and their Supernatants.

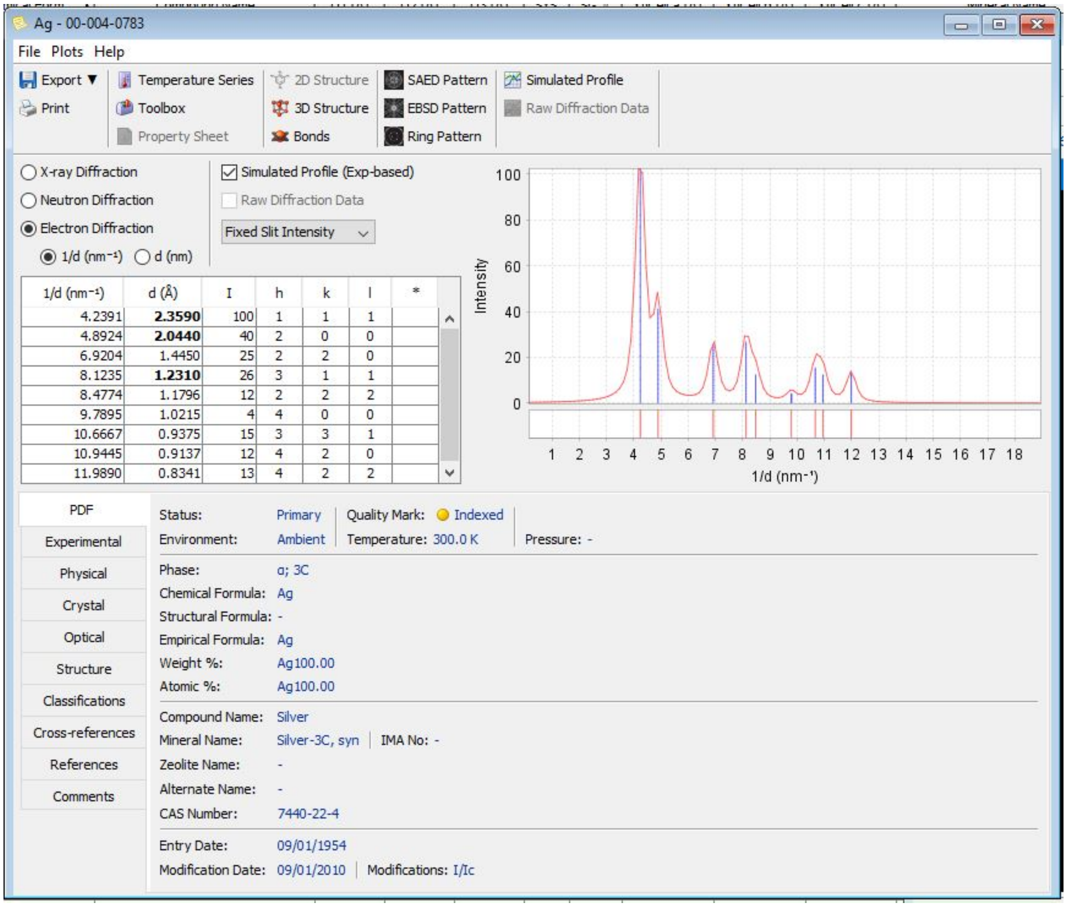

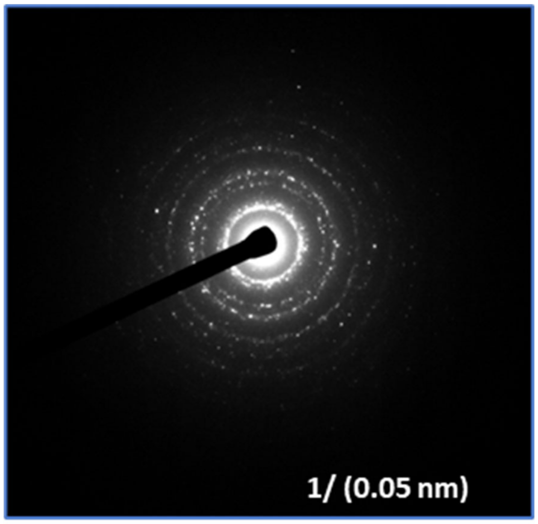

Figure S2. SAED pattern of Ag-NPs synthesised from *T. ferdinandiana* extracts.

Table S2. Average (Av) percentage inhibition of *E. coli, S. aureus, P. aeruginosa* and *P. mirabilis* at different concentrations (mg/mL). All values were expressed as mean ± SD of three replicates.

E. coli	*AgKL*		*AgKP*		*AgKPL*		*KL*		*KP*		*KPL*	
Concentration (mg/mL)	%	SD	%	SD	%	SD	%	SD	%	SD	%	SD
0.16	58.8	1.2	29.5	1.8	99.1	1.4	28.9	1.4	22.4	2.8	24.4	9.1
0.31	97.2	5.9	39.1	3.7	92.5	2.2	31.7	3.5	30.1	1.9	31.8	3.5
0.63	101.3	1.3	45.8	4.3	100.7	3.1	41.8	3.0	31.3	1.6	34.5	2.4
1.25	99.9	2.8	50.2	2.3	92.4	4.0	59.1	1.0	34.9	1.7	45.4	5.1
2.50	95.9	2.1	50.2	4.8	102.2	2.1	61.1	1.7	50.1	3.4	58.5	3.5
5.00	105.0	5.4	47.9	2.8	93.5	2.5	57.5	2.2	63.0	15.5	66.9	5.7

S. aureus	*AgKL*		*AgKP*		*AgKPL*		*KL*		*KP*		*KPL*	
Concentration (mg/mL)	%	SD	%	SD	%	SD	%	SD	%	SD	%	SD
0.16	51.5	3.5	37.7	4.2	68.5	4.4	28.8	4.7	28.2	2.0	25.2	3.8
0.31	41.2	6.4	34.3	6.3	57.6	5.5	30.2	3.5	27.2	2.4	27.1	4.9
0.63	78.8	3.4	56.0	8.0	87.4	7.2	39.0	2.8	59.4	3.7	45.0	4.7
1.25	103.9	7.4	80.0	19.5	68.6	3.3	70.9	3.5	97.5	1.2	96.6	0.6
2.50	90.0	7.9	53.6	6.3	106.5	6.7	91.5	1.3	96.8	0.8	95.8	1.8
5.00	109.6	4.7	50.4	4.8	91.9	6.5	89.1	1.2	96.7	0.5	91.3	12.8

P. aeruginosa	*AgKL*		*AgKP*		*AgKPL*		*KL*		*KP*		*KPL*	
Concentration (mg/mL)	%	SD	%	SD	%	SD	%	SD	%	SD	%	SD
0.16	28.1	2.3	19.7	5.7	46.5	3.6	21.5	2.6	15.7	1.4	16.8	2.2
0.31	63.4	2.9	33.7	8.9	74.0	8.3	23.6	1.9	19.9	1.0	22.4	2.6
0.63	97.7	4.1	50.4	1.8	72.4	9.6	25.2	2.5	22.0	1.2	25.6	2.5
1.25	104.3	4.1	53.8	3.5	82.3	7.8	29.0	2.5	22.5	4.0	24.6	1.6
2.5	98.1	1.9	59.2	2.1	97.5	5.4	27.7	2.8	18.0	2.1	22.7	4.2
5	107.8	4.1	64.9	4.9	97.8	5.9	30.6	7.2	16.8	3.2	34.6	15.4

P. mirabilis	*AgKL*		*AgKP*		*AgKPL*		*KL*		*KP*		*KPL*	
Concentration (mg/mL)	%	SD	%	SD	%	SD	%	SD	%	SD	%	SD
0.16	56.9	3.4	50.7	1.9	63.1	9.0	51.0	2.4	50.1	1.2	49.2	2.0
0.31	73.3	4.7	57.7	1.7	90.5	4.3	56.8	2.8	51.4	1.0	55.2	1.4

0.63	101.2	1.6	61.7	1.6	83.8	3.4	60.1	1.6	54.7	2.8	58.5	1.0
1.25	96.6	1.4	61.9	3.1	89.6	7.2	63.6	1.3	55.5	2.5	59.0	0.7
2.5	98.0	3.1	63.8	5.1	83.3	7.0	63.6	2.4	60.7	2.4	62.3	2.5
5	112.0	7.1	66.9	4.8	103.6	7.2	64.7	2.3	66.4	2.9	72.7	2.0

References

1. Ali, N.B.; Ibrahim, S.S.A.; Alsherbiny, M.A.; Sheta, E.; El-Shiekh, R.A.; Ashour, R.M.; El-Gazar, A.A.; Ragab, G.M.; El-Gayed, S.H.; Li, C.G.; et al. Gastroprotective potential of red onion (Allium cepa L.) peel in ethanol-induced gastric injury in rats: In-volvement of Nrf2/HO-1 and HMGB-1/NF-κB trajectories. J. Ethnopharmacol. 2024, 319, 117115. https://doi.org/10.1016/j.jep.2023.117115
2. Fraisier-Vannier, O.; Chervin, J.; Cabanac, G.; Puech, V.; Fournier, S.; Durand, V.; Amiel, A.; André, O.; Benamar, O.A.; Dumas, B.; et al. MS-CleanR: A Feature-Filtering Workflow for Untargeted LC–MS Based Metabolomics. *Analytical Chemistry* **2020**, *92*, 9971-9981, doi:10.1021/acs.analchem.0c01594.
3. Tsugawa, H.; Kind, T.; Nakabayashi, R.; Yukihira, D.; Tanaka, W.; Cajka, T.; Saito, K.; Fiehn, O.; Arita, M. Hydrogen Rearrangement Rules: Computational MS/MS Fragmentation and Structure Elucidation Using MS-FINDER Software. *Analytical Chemistry* **2016**, *88*, 7946-7958, doi:10.1021/acs.analchem.6b00770.
4. Alsherbiny, M.A.; Bhuyan, D.J.; Radwan, I.; Chang, D.; Li, C.-G. Metabolomic identification of anticancer metabolites of Australian propolis and proteomic elucidation of its synergistic mechanisms with doxorubicin in the MCF7 cells. *International Journal of Molecular Sciences* **2021**, *22*, 7840.
5. Ogaly, H.A.; Alsherbiny, M.A.; El Badawy, S.A.; Abd-Elsalam, R.M.; Li, C.G.; Azouz, A.A. Gastroprotective effects and metabolomic profiling of Chasteberry fruits against indomethacin-induced gastric injury in rats. *Journal of Functional Foods* **2021**, *86*, 104732.
6. Dührkop, K.; Fleischauer, M.; Ludwig, M.; Aksenov, A.A.; Melnik, A.V.; Meusel, M.; Dorrestein, P.C.; Rousu, J.; Böcker, S. SIRIUS 4: a rapid tool for turning tandem mass spectra into metabolite structure information. *Nature methods* **2019**, *16*, 299-302.
